# Identification of Cyanobacteria in a Eutrophic Coastal Lagoon on the Southern Baltic Coast

**DOI:** 10.3389/fmicb.2017.00923

**Published:** 2017-05-29

**Authors:** Martin Albrecht, Thomas Pröschold, Rhena Schumann

**Affiliations:** ^1^Applied Ecology and Phycology, University of RostockRostock, Germany; ^2^Research Institute for Limnology, University of InnsbruckMondsee, Austria; ^3^Applied Ecology and Phycology, Biological Station Zingst, University of RostockRostock, Germany

**Keywords:** Cyanobacteria, *Aphanothece*, Cyanobium, lagoon, brackish water, phytoplankton, 16S rRNA

## Abstract

Cyanobacteria are found worldwide in various habitats. Members of the picocyanobacteria genera *Synechococcus* and *Prochlorococcus* dominate in oligotrophic ocean waters. Other picocyanobacteria dominate in eutrophic fresh or brackish waters. Usually, these are morphologically determined as species of the order Chroococcales/clade B2. The phytoplankton of a shallow, eutrophic brackish lagoon was investigated. Phytoplankton was dominated by *Aphanothece*-like morphospecies year-round for more than 20 years, along a trophy and salinity gradient. A biphasic approach using a culture-independent and a culture-dependent analysis was applied to identify the dominant species genetically. The 16S rRNA gene phylogeny of clone sequences and isolates indicated the dominance of *Cyanobium* species (order Synechococcales sensu Komárek/clade C1 sensu Shih). This difference between morphologically and genetically based species identifications has consequences for applying the Reynolds functional-groups system, and for validity long-term monitoring data. The literature shows the same pattern as our results: morphologically, *Aphanothece*-like species are abundant in eutrophic shallow lagoons, and genetically, *Cyanobium* is found in similar habitats. This discrepancy is found worldwide in the literature on fresh- and brackish-water habitats. Thus, most *Aphanothece*-like morphospecies may be, genetically, members of *Cyanobium*.

## Introduction

Cyanobacteria live in most habitats worldwide and are important primary producers. Cyanobacteria often dominate the phytoplankton under meso- or eutrophic conditions. Extensive summer blooms are well known from the Baltic Sea (Wasmund, 1997; Stal et al., 2003) and freshwater lakes (Komárková-Legnerová and Cronberg, 1994; Willame et al., 2005; Michalak et al., 2013). Very small single-cell cyanobacteria (< 2 μm; picocyanobacteria = APP) are most abundant in the oligotrophic open oceans. Most of these are assigned as species of *Synechococcus* and *Prochlorococcus* (Chisholm et al., 1992). Other, but closely related, *Synechococcus*-like cyanobacteria succeed in eutrophic coastal waters (Caroppo, 2015 and references therein). The brackish waters are relatively little investigated so far (Celepli et al., 2017), but also show high abundances of APP and colonies with cells <2 μm. Thus, different cyanobacteria inhabit and can sometimes dominate almost all aquatic systems, even under highly contrasting conditions. Interestingly, most of these cyanobacteria are only assigned as *Synechococcus*-like organisms, knowing that the genus *Synechococcus* is polyphyletic (see Komárek et al., 2014 and references therein).

Monitoring programs target cyanobacterial blooms especially, because of their potential toxicity and high biomass following eutrophication (HELCOM, 1988). The degradation of the bloom biomass may deplete oxygen, and diazotrophic cyanobacteria have a strong influence on nutrient cycles (Larsson et al., 2001). Up to the present, monitoring by environmental agencies has relied on determinations based on morphotypes. Monitoring within the Baltic Sea region refers to two databases: AlgaeBase^1^ (Guiry and Guiry, 2016. AlgaeBase. World-wide electronic publication, National University of Ireland, Galway) and WoRMS World Register of Marine Species^2^ (WoRMS Editorial Board, 2016). One drawback of the WoRMS database is its focus primarily on marine species, which may not cover all species of brackish systems. Another problem in using these databases is that they may not be congruent with the most recent taxonomic results.

**FIGURE 1:**
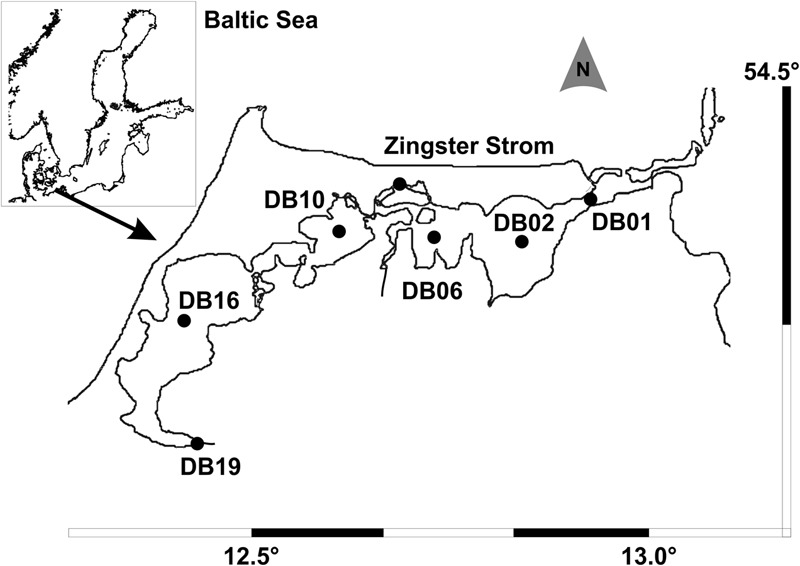
**Inner coastal lagoon system Darß-Zingst Bodden Chain with the sampling stations (modified from Schumann et al., 2006)**.

In some shallow turbid waters, cyanobacteria produce high and stable biomasses, which maintain poor underwater light availability. Examples of these aquatic systems include two lagoons in the northern Adriatic (Sorokin et al., 2004, 2006), Laguna Chascomús in Argentina (Iachetti and Llames, 2015) and the Curonian Lagoon in the southern Baltic (Olenina, 2013). Two German Baltic lagoons are the Darß-Zingst Bodden chain (DZBC) and the inner Rügenian Bodden (**Figure 1**), which are also dominated by very small, coccoid and gelatinous colony-forming cyanobacteria (Schumann et al., 2001). Even these very small non-heterocytous picocyanobacteria can be capable of nitrogen fixation, e.g., *Aphanothece* (Singh, 1973; Bergmann et al., 1997). However, the colonies of very small cells, solitary cyanobacteria and green algae in the picoplankton range (<2 μm) are often overlooked or may be assigned to the wrong taxonomic groups.

In accordance with morphological data, i.e., solitary rod-shaped cyanobacteria <2 μm in length, the genera *Synechococcus* and *Prochlorococcus* were found using molecular approaches in picoplankton-dominated waters (Chisholm et al., 1992; Robertson et al., 2001; Rocap et al., 2003; Chen et al., 2006; Caroppo, 2015). Geiß et al. (2004) also found *Synechococcus* 16S rRNA gene sequences in the DZBC, rather than *Aphanothece* sequences as suggested by morphology. Therefore, the identity of the most abundant cyanobacteria remained uncertain.

The identification of cyanobacteria based on morphology is dependent on easily visible features that are easy to recognize, even at lower magnifications with light microscopy. This is often very difficult, especially for groups without such features and with small cell sizes. In addition, often very little is known about their phenotypic plasticity. For example, in picocyanobacteria, unicellular specimens smaller than 2–3 μm were assigned as species of *Synechococcus* or *Cyanobium*, whereas specimens in the same size range forming colonies and mucilage were identified as species of *Aphanothece, Aphanocapsa* or *Anathece*. Jezberová and Komárková (2007a) and Huber et al. (2017) demonstrated that *Cyanobium* species can form colonies under grazing pressure, which then morphologically resemble *Anathece*. Komárek et al. (2011) confirmed that these two genera are closely related, based on 16S rRNA phylogeny.

Only a few morphological features at the cellular level are characteristic for the basal orders Gloeobacterales and Synechococcales, but also occur in Chroococcales (Komárek et al., 2014). This leads to confusion in the identification of simple coccoid cyanobacteria. Species of higher orders have a wider morphological plasticity and can show special features such as branched trichomes, heterocytes or akinetes (Komárek et al., 2014). Many species of simple coccoids cannot be identified by light microscopy, so there may be an enormous cryptic diversity (Komárek et al., 2014) or mis-interpreted results. Therefore, it is not possible to form conclusions on the capabilities of cyanobacterial communities based on morphology alone (Tanabe et al., 2009; Palinska and Surosz, 2014; Komárek, 2016).

To solve this problematic situation in cyanobacteria, molecular data have been used to determine cyanobacteria (e.g., Palinska and Surosz, 2014); however, sequencing is not common practice in monitoring by environmental agencies because of lack of facilities and financial support. In contrast, the scientific communities have the facilities and perhaps the finances to perform the sequencing, but do not have the ability to practice this at larger scales.

Therefore, the aim of this study was to connect the scientific with the applied standards, to address both communities and to provide solutions for filling this gap, e.g., acknowledging both the microscopy and the genetic determination via polyphasic approaches. We identified the dominant planktonic cyanobacteria of the DZBC, using both culture-dependent and -independent approaches. The following questions were addressed: (i) Which genetic lineages of cyanobacteria dominate in the DZBC? (ii) Do the long-term monitoring data reflect the genetic data? (iii) What do the findings mean for monitoring data from other lagoons? To answer these questions, we wanted to determine isolates by both morphological and molecular genetic methods, to acquire a deeper insight into the phylogeny of the dominant organisms. From the results, we hoped to obtain some information about the influence of these organisms on the ecosystem, as well as on the applicability of the Reynolds functional classification of freshwater phytoplankton (Reynolds et al., 2002), which is a standard tool for microscopy-based classification.

## Materials and Methods

### Sampling Site, Collecting, Culture and Microscopy

The sampling site was the Darß-Zingst Bodden Chain (**Figure 1**), a brackish lagoon system of four basins linked by narrow streams, with both a salinity gradient (2–12) and a trophy gradient. Phytoplankton samples have been counted for one site (Zingster Strom) since 1991 at weekly (summer) to biweekly (winter) intervals. Additional samples from the trophy gradient are investigated occasionally (1996: Schumann et al., 2001, 2006; this study, 2015: not yet available). Therefore, the annual cycle of the Zingster Strom is given for 2006, for comparison with the species composition in the trophy gradient from the same year. Samples for molecular analyses were taken in spring 2013 at the Zingster Strom. Spring phytoplankton biomass and composition was estimated for 11 samples.

The Zingster Strom is a eutrophic site between the two innermost water basins, with an average salinity of 5.6 (Schumann et al., 2006) and high turbidity. In 2006, salinity was 5.3, near the long-term average. The mean salinity in 2013 was below average, 4.8 (unpublished data). During sampling in April and May 2013 (clones, most of the isolates), salinity ranged from 2.8 to 5.9. This was a period with no extreme values. The water column was always well mixed, and the samples were taken at the water surface. Five hundred milliliter was concentrated by centrifugation at 10,000 rpm (BiofugePico, Heraeus) to 2 ml for DNA extraction (see below). Samples for cell counts were fixed with Lugol (5 drops per 20 ml) and stored in darkness. Samples for epifluorescence microscopy were preserved with 1 ml 25% glutaraldehyde per 20 ml sample and stored refrigerated.

Uni-cyanobacterial cultures were established directly from the plankton samples. First, 200 μl of the Bodden sample was plated on BG11 (Rippka and Herdman, 1992) soft agar plates (0.75% w/v agar, Difco^®^) and incubated for 4 weeks at 20°C with a light:dark cycle of 16:8 h at 40 μmol photons m^-2^ s^-1^ (Osram Lumilux Deluxe Daylight). The plates were checked weekly under a binocular stereomicroscope (Olympus SZ40). Colonies growing on the plates were distinguished by color, then isolated with a sterilized toothpick and transferred to new agar plates. Upon transfer into liquid medium, the samples were checked again, using a light microscope (Olympus BX-51) with a magnification of up to 100×. Microphotographs were taken with a 100× oil immersion objective, Colorview12 camera and AnalySiS (Soft Imaging Solutions, Olympus), and later with the Olympus UC-30 camera and CellSens Entry software package (Olympus). Colonies were stained with India Ink to observe mucilage. Phytoplankton and isolates were determined using mainly the Süßwasserflora von Mitteleuropa 19/1–3 (Komárek and Anagnostidis, 1999, 2005; Komárek, 2013). Additionally, the Algenflora der Ostsee (Pankow, 1990) was used as a local taxonomic key. All species names were checked for current status and names.

### Cell Counts

Small cyanobacteria were investigated by epifluorescence microscopy. Subsamples of 0.5–1 ml were filtered (-100 mbar) onto Irgalanblack stained polycarbonate Track-Etch membranes (Sartorius, 0.2 μm, 25 mm). They were mounted with 1 ml DAPI solution (freshly filtered through 0.2 μm cellulose acetate, 92 μmol DAPI l^-1^ in phosphate buffer pH = 7.6) for 5 min. The stain was removed also by filtration and the filter was mounted between a slide and cover slip with thin layers of immersion oil (Olympus, fluorescence-free). DAPI staining would not have been necessary for counting only cyanobacteria, but the same filters were used for heterotrophic bacteria (not shown here). However, some samples showed only weak autofluorescence (mostly in May and June), where the cells were also checked under UV excitation (U-MWU2).

Usually, the green excitation filter cube (U-MWG2) was used (Olympus BX51, UPlan FL 100 NA 1.3 oil objective). At least 200 cells were counted per filter (in at least 20 ocular grids of 10,000 μm^2^) resulting in a confidence range of ±14% (Poisson distribution). Regularly, replicate filters were checked and the standard deviation was <20%. Several colony types were checked for cells per colony. The “*Aphanothece*” complex unites all colonies with rod-shaped cells, e.g., *Cyanodictyon, Cyanonephron, Lemmermaniella* and *Aphanothece* in the sense of morphotypes. The complex of *Snowella* and *Woronichinia* (formerly *Gomphosphaeria*) contains all colonies of larger (>2 μm diameter) cells of spherical or ovoid shape with gelatinous stalks. Ten colonies per sample were counted for their cells. The average (median) cell number per colony was calculated for monthly averages (Zingster Strom, *n*_colony_ = 30–40) or for quarters (all other sites, *n*_colony_ = 30). Total cells were calculated by multiplying colony number with average cells per colony.

Colonies and eukaryotic phytoplankton was counted in sedimentation chambers (volume 1 ml, depth 3 mm) under a laboratory light microscope (Euromex) at a magnification of 256× (16× oculars and 16× objective). At least 18 ocular grids of 0.36 mm^2^ were counted along the chamber diameter. More than 100 individuals were counted for 3 dominant species. Alternatively, >500 individuals were counted in total if there were not enough dominant species (Helcom, Combine manual). Replicate counts were not done. Cell sizes of small cyanobacteria were determined by scanning electron microscopy after critical-point drying, filament width was measured in fresh samples by image analysis. Filament lengths were all measured upon counting. Larger eukaryotes, especially diatoms, were counted in size classes.

### Molecular Analyses

DNA was extracted from isolates with the Qiagen DNeasy Plant Mini Kit following the manufacturer’s manual. The only changes in the protocol were a longer period (1 h) on the heating block, and omission of a second elution step. The time on the heating block was increased to obtain larger amounts of DNA.

For clone libraries, the 16S rRNA gene of the extracted DNA from the whole DZBC samples was amplified in a PCR reaction using the primers CYA106F and CYA781R (Nübel et al., 1997) for clone libraries. The PCR products of the isolated strains were amplified using the Qiagen Taq-polymerase Master Mix and cyanobacteria-specific primers (SSU-4-forw and CPL-10R; Wilmotte et al., 1993; Marin et al., 2005). For both PCRs a Biometra Thermocycler was used, with the following program: 3 min initial denaturation at 96°C followed by 30 cycles of 1 min at 96°C (denaturation), 2 min at 55°C (annealing), 3 min at 68°C (elongation), and a final elongation step of 10 min.

A subsample of the PCR products of isolates and clones was checked for bands via gel electrophoresis on 2% Agarose (Biozym) gels. Gel electrophoresis was carried out for 1 h at 120 V. Gels were prepared with 0.5× Tris Borate EDTA-Buffer (TBE, Sigma–Aldrich) and Midori Green Advanced (Nippon Genetics). Gel bands were checked and documented under UV-light (UVP DocIt^®^), and then with FastGene GelPic LED Box (Nippon Genetics). PCR products were purified using the Qiagen PCR Purification Kit and PCR Purification Kit by Macherey-Nagel. Both kits were used following the manufacturer’s manual. Purified products were then transferred to a 96-well sequencing plate and mixed with sequencing primers. Sequencing was done by the Qiagen Sequencing Service.

All sequences of each strain were assembled automatically using the Geneious^TM^ software package. Assembled contigs were checked manually for consistency of overlapping regions. Complete contigs were compared to existing entries in the NCBI data base using BLASTn-Search (Altschul, 1990). All contigs were then aligned using BioEdit (Hall, 1999) and SeaView (Gouy et al., 2010). A basis for alignment was the secondary structure of the 16S rRNA. Therefore, variable helices were folded using the mfold web server (Zuker, 2003) and conserved structure elements were taken as a basis for homolog regions. Reference sequences for phylogenetic analysis were taken from Shih et al. (2013) and several sequences directly from NCBI. They were selected for most comprehensive information, i.e., availability of an isolate, inclusion into a publication, sequence length and sequence quality.

16S rRNA gene phylogenies were calculated using Maximum Likelihood (ML) in MEGA 6 (Tamura et al., 2013), in RAxML (Stamatakis et al., 2005) and MrBayes for bayesian analysis. The MEGA 6 implemented Modeltest for best RNA/DNA model was run first, and the model with lowest l nL in the Akaike Criterion (AIC; Akaike, 1981) was chosen for Maximum Likelihood (ML) analysis. As an out-group, *Gloeobacter violaceus* PCC 7421 was used. The phylogeny clades were checked for reference strains and assigned to the clade names of Shih et al. (2013) and Komárek et al. (2014).

## Results

### Abundance and Composition

**FIGURE 2:**
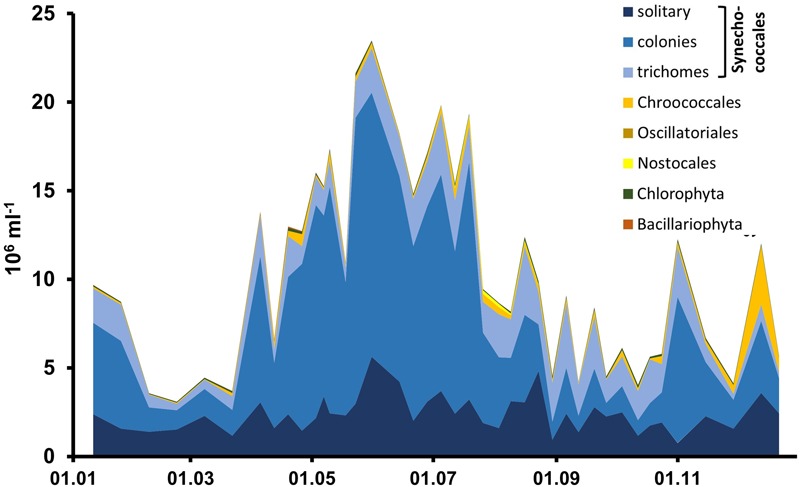
**Phytoplankton abundance based on counts with the light microscope, for the year 2006**.

**FIGURE 3:**
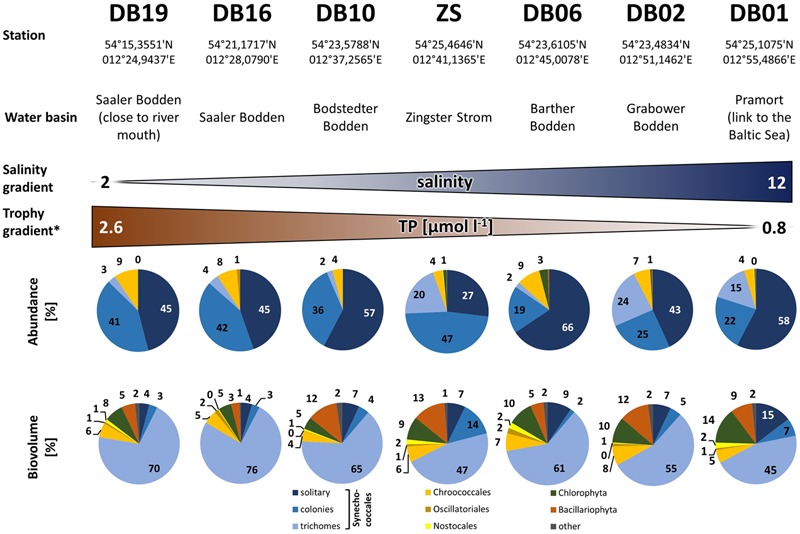
**Phytoplankton abundance and biovolume along the salinity and nutrient gradients of the Darß-Zingst Bodden Chain.** The diagrams show the annual mean composition (*n* = 12 for DB-stations and *n* = 52 for station ZS) of the year 2006. ^∗^More detailed data in Supplementary Material.

Cyanobacteria of the orders Synechococcales and Oscillatoriales were always dominant in biomass and abundance, as shown in detail for the year 2006 for the Zingster Strom (**Figure 2**) and the 2006 yearly means for the whole lagoon system (**Figure 3**). Phytoplankton community showed nearly the same patterns and species composition over the last 20 years. Spring and summer biomasses differed from autumn blooms. In spring, Synechococcales and Bacillariophyceae dominated phytoplankton biomass. In autumn, cyanobacterial colonies and trichomes formed more than 80% of biomass. Synechococcales were present with high biomass and abundance the whole year (**Figure 2**). Chlorophyceae and Bacillariophyceae were also always present in phytoplankton, but mostly with low biomass. The dominance of certain species (morphotypes) varied from year to year, but cyanobacteria were always dominant. The dominance of cyanobacteria in abundance and biomass is found in the whole DZBC over the salinity and nutrient gradients that exist there (**Figure 3**).

**FIGURE 4:**
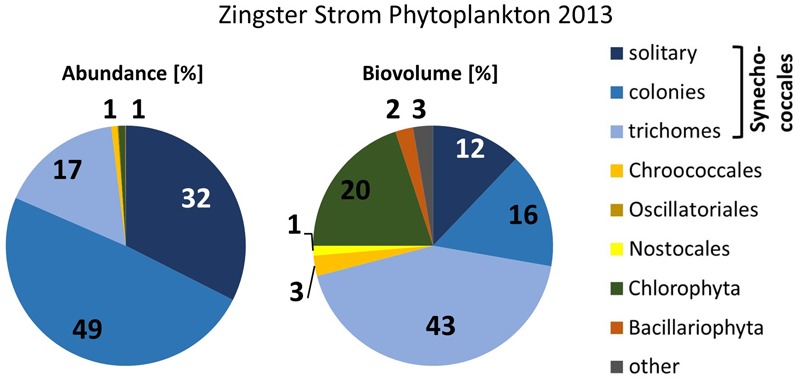
**Phytoplankton abundance and biovolume at the Zingster Strom, based on counts with the light microscope.** Mean percentages for spring 2013 (*n* = 11).

The very small colony forming species (Synechococcales, *Cyanobium*) appeared with 2–21.10^6^ cells ml^-1^ (**Figure 2**) and comprised 47–93% of phytoplankton biomass throughout the year 2006. Cyanobacteria with heterocytes were present, but did not play a major role regarding phytoplankton biomass (<2%). Toxic species such as *Microcystis, Nodularia, Cylindrospermopsis* and *Anabaena* played even minor role in the DZBC. They were only recognized in net samples for zooplankton (concentrated fivefold). All other species had an abundance of <0.23.10^6^ cells ml^-1^. Biomass and abundance of the Zingster Strom phytoplankton was also dominated by colony-forming picocyanobacteria during the sampling period in April and May 2013 (**Figure 4**). The trichomes of *Planktolyngbya* showed the highest biomass, while colonies and single cells of Synechococcales showed the highest abundance.

**FIGURE 5:**
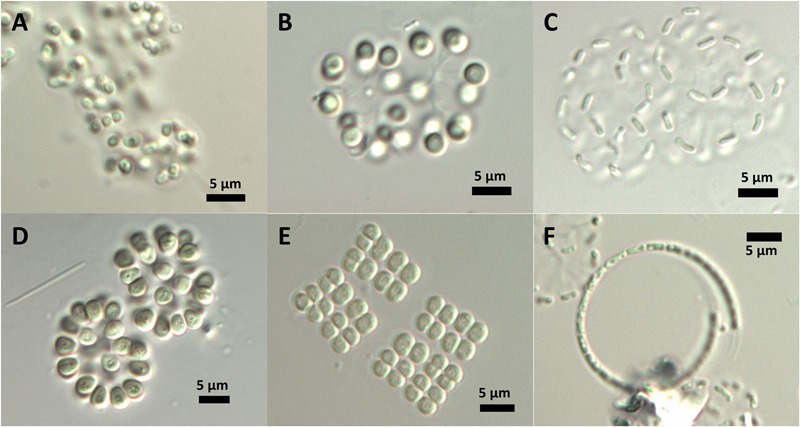
***In situ* morphology of DZBC cyanobacteria species. (A)** *Gloeothece* sp.; **(B)** *Snowella* sp.; **(C)** *Cyanodictyon* sp.; **(D)** *Woronichinia* sp.; **(E)** *Merismopedia* sp.; **(F)** *Planktolyngbya contorta.*

Most cyanobacteria of the DZBC had thick mucilage and formed colonies. The most abundant morphotypes belonged to the *Aphanothece*-complex, as described by Pankow (1990) for the genus. The cells were 1–2 μm long, rod-shaped, and straight (*Aphanothece*) or curved (*Cyanodictyon*). Colonies were compact (*Aphanothece*) or in loose formation (*Cyanodictyon*). Other cells were oval to spherical (*Snowella, Merismopedia, Chroococcus, Aphanocapsa, Gloeothece*) and formed regular (*Merismopedia, Chroococcus*) or irregular colonies (*Snowella, Aphanocapsa*) (**Figure 5**). Genus and species determination depended mostly on the colony shape. Cells were often too small to recognize features within the cells, if present. Only genera of *Snowella, Woronichinia, Merismopedia* and *Microcystis* were determined to species level at counting magnification, by their distinct morphotypes. *Aphanothece, Aphanocapsa* and *Cyanodictyon* were determined only at genus level, because their very small cells could hardly be measured to determine species. Non-colony-forming APP (autotrophic picoplankton) single cells were usually rod-shaped and were counted as *Synechococcus* sp.

The different cyanobacterial species are used to infer the ecological status of the water. One system for water classification is the Reynolds functional group system (Reynolds et al., 2002). In the Reynolds functional group system, this phytoplankton community fitted best into the K group. The habitat specification of this group is shallow and nutrient-rich, and typical representatives are *Aphanothece* and *Aphanocapsa* (Reynolds et al., 2002). The significance of this group’s community structure was uncertain, except for a high pH tolerance (Reynolds et al., 2002).

### Morphological and Molecular Identification of Cyanobacterial Species

**FIGURE 6:**
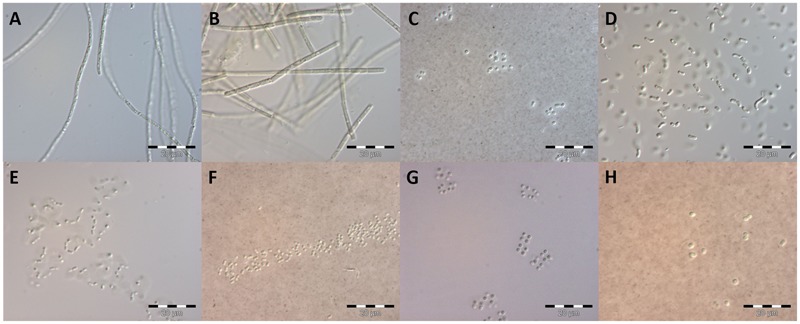
**Early culture stage (<4 weeks) of isolates as basis for morphological species determination.** Species names refer to the morphological determination. Photographs **(C,F,H)** were stained with India Ink. **(A)** *Limnothrix* cf. *obliqueacuminata;* **(B)** *Limnothrix* cf. *rosea;* **(C)**: *Aphanothece* cf. *nidulans;* **(D)**: *Rhabdoderma* cf. *linearis;* **(E)**: cf. *Cyanodictyon planctonicum;* **(F)**: cf. *Aphanocapsa incerta;* **(G)**: cf. *Chroococcus vacuolatus;* **(H)**: *Synechocystis* cf. *diplocca*; scale bars: 20 μm.

**FIGURE 7:**
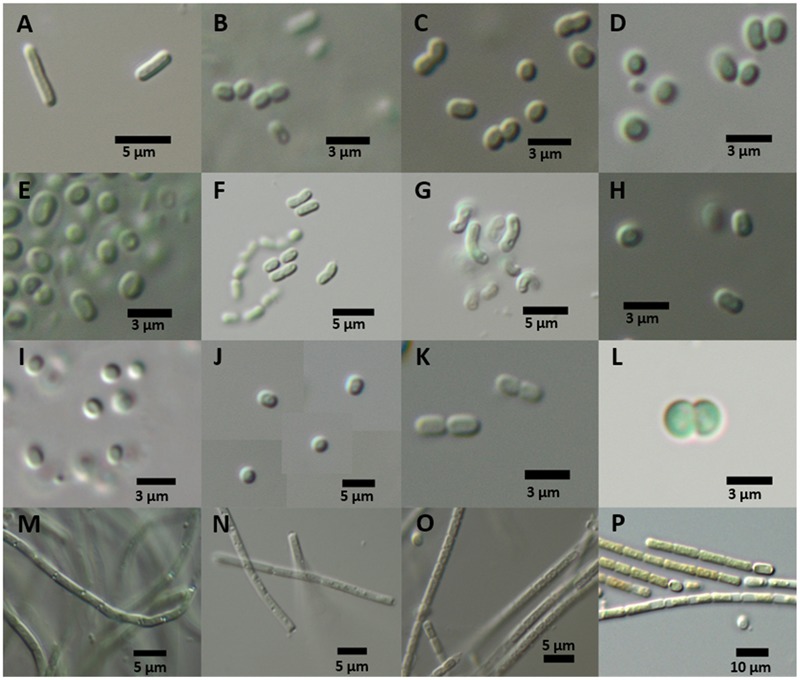
**Culture morphology of representative strains of phylogenetic clades after 1 year of culture. (A)** Shows *Synechococcus elongatus* type strain and **(B–P)** show isolates of the DZBC. Generic names refer to the 16S rRNA gene phylogeny position. CZS-strains were isolated from the Zingster Strom. RK- and SB-strains originate from the Saaler Bodden (station DB16). **(A)** *Synechococcus elongatus* PCC 6301; **(B–K)** DZBC isolates of *Cyanobium*; **(B)** CZS 19G; **(C)** CZS 39E; **(D)** CZS 22C; **(E)** CZS 25K; **(F)** CZS 48K; **(G)** CZS 48M; **(H)** CZS 25G; **(I)** CZS 27J; **(J)** CZS 34G; **(K)** CZS 45I; **(L)** *Merismopedia punctata* strain CZS 2/3; **(M)** *Leptolyngbya* strain RK 2.3; **(N)** *Pseudanabaena* strain CZS 36B; **(O)** *Pseudanabaena* strain CZS 45C; **(P)** Nostocales strain SB 3.21; scale bars: 3 μm **(B–E,H,I,K,L)**; 5 μm **(A,F,G,J,M–O)**; 10 μm **(P)**.

The isolation of strains in a culture-dependent approach was a first attempt at more-precise identification and investigation of DZBC cyanobacteria. Fifty-three isolates were established. These isolates were determined morphologically as *Limnothrix* cf. *obliqueacuminata, Limnothrix* cf. *rosea, Aphanothece* cf. *nidulans, Rhabdoderma* cf. *linearis*, cf. *Cyanodictyon planctonicum*, cf. *Aphanocapsa incerta*, cf. *Chroococcus vacuolatus, Synechocystis* cf. *diplocca* (**Figure 6**), *Cyanobium* sp., *Pseudanabaena* sp., *Merismopedia punctata* and *Cronbergia* sp. (**Figure 7**). Morphology changed during culturing, from gelatinous aggregates to single cells (**Figures 6, 7**). Strain CZS 2/3 (*Merismopedia punctata*) was the only isolate that was determined *in situ*. Therefore, this species determination is based on its original morphology and is the most reliable. The other isolates were named later, by their phylogenetic affiliation.

**FIGURE 8:**
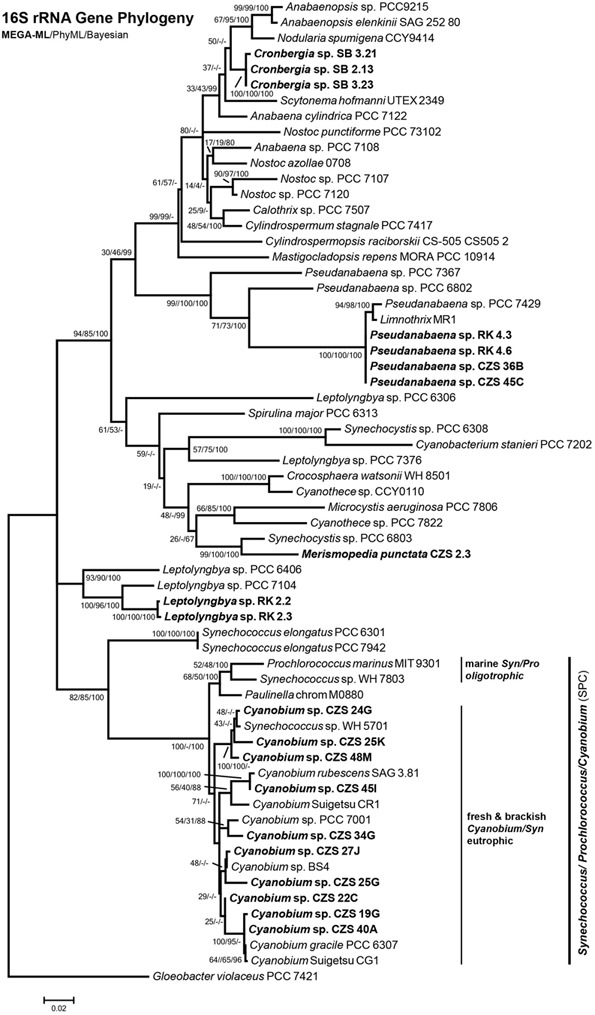
**Maximum Likelihood Phylogeny constructed with MEGA of 16S rRNA gene sequence (1356 bp).** Node numbers show MEGA ML/PhyML/Bayesian statistical bootstrap support. In bold: DZBC isolates. Clade names sensu Shih et al. (2013) and Komárek et al. (2014).

The isolates were taken for genetic analysis of the complete 16S rRNA gene, available under accession numbers KY379855–KY379897. First, the sequences were checked with BLASTn. The best hit of BLASTn assigned isolates to the genera *Synechococcus, Cyanobium, Pseudanabaena* and *Merismopedia.* Some isolates could not be identified using BLASTn, because the nearest database sequence differed by more than 3% in nucleotides. These isolates belonged to the order Nostocales, after Komárek et al. (2014), which is clade B1 (Shih et al., 2013). The morphology also indicated Nostocales (clade B1) strains (**Figure 7**). According to the 16S rRNA gene phylogenies, all isolates belonged to the orders Nostocales (clade B1), Chroococcales (clade B2) or Synechococcales (clades C1 and F) (**Figure 8**). The isolates were all assigned to one of 5 clades, treated as genera [1 genus of Nostocales, 1 genus of Chroococcales and 3 genera of Synechococcales (1 genus in clade C1, 1 genus in clade C3 and 1 genus in clade F)]. Most isolates were part of the clades of *Cyanobium* (44; clade C1) and *Pseudanabaena* (9; clade F) (Synechococcales). Two isolates belonged to *Leptolyngbya* (clade C3; Synechococcales) and one isolate was part of the *Merismopedia*-clade (clade B2; Chroococcales). Three isolates were assigned to the order Nostocales (clade B1). The phylogeny did not show a clear assignment to a clade within Nostocales, and database searches showed the highest nucleotide congruency with only 97% sequence identity. The new clade is closely related to *Cronbergia* strain PCC6471 species. Morphology and molecular data showed contrasting results for the very small isolates. Morphologically, most isolates were determined as genera and species of the order Chroococcales. However, phylogenetically, they belong to the order Synechococcales (clade C1).

The heterocytous and non-heterocytous filamentous strains were mostly isolated from samples taken in autumn. Filamentous cyanobacteria occurred with the highest biomasses in that season (Schumann et al., 2001; Schoor et al., 2008). Only the very abundant non-heterocytous *Planktolyngbya contorta* could not be cultured. Most filaments did not have heterocytes and belonged to the orders Synechococcales (clade F) and Oscillatoriales (clade C3). Synechococcales filaments were morphologically assigned to the genera *Pseudanabaena* and *Limnothrix.* Other genera were determined as *Leptolyngbya* (clade C3) and Nostocales (clade B1) genus *Cronbergia*.

A second attempt was undertaken for a preliminary overview of the genotypes present. The clones of a full DZBC sample resulted in 52 sequences (KY327309-KY327360). Six genus names were found with BLAST first hits: *Synechococcus, Cyanobium, Cyanobacterium, Aphanocapsa, Snowella* and *Limnococcus*. The “*Synechococcus”* and *“Cyanobacterium”* clone sequences were quite similar (99%) to *Cyanobium* PCC7001 within the clade C1/Synechococcales. Therefore, this result was partly in accordance with the *in situ* morphotypes. A phylogenetic analysis with selected sequences of the 608 bp fragment of the 16S rRNA gene showed the same results. Four genera were found based on the 16S rRNA gene phylogeny positions. Most clones belonged to the clades *Snowella* (22 clones; clade B2/ Chroococcales) and *Cyanobium* (20 clones, clade C1, Synechococcales).

## Discussion

### Morphology *In Situ*

Former investigations on the DZBC focused on the morphological determination of the cyanobacterial phytoplankton. Most abundant genera were *Snowella, Planktolyngbya, Aphanothece, Aphanocapsa, Dolichospermum, Aphanizomenon, Coelosphaerium, Chroococcus, Merismopedia, Microcystis, Nodularia* and *Limnothrix* (Pankow and Martens, 1973; Kell, 1973; Pankow, 1976; Nasev et al., 1978; Schumann et al., 2005). Pankow (1976) found about 40 different species per sampling station over the entire DZBC. The overlap of species between stations results in 61 cyanobacterial species in total for the entire DZBC. In later studies, Klinkenberg and Schumann (1995) found non-colony-forming APP picocyanobacteria, which were determined genetically as *Synechococcus*-like cyanobacteria (Geiß et al., 2004).

The small-celled species (0.8–2 μm) occurred in very high cell numbers and also high biomass over the year. Other brackish waters with described *Aphanothece*-like species are the Curonian Lagoon and Vistula Lagoon, which are also located on the southern Baltic coast. However, filamentous, heterocytous cyanobacteria and *Microcystis* were most abundant in those lagoons (Pilkaitytë, 2007; Paldavičienė et al., 2009; Aleksandrov, 2010; Lange, 2011, 2014; Nawrocka and Kobos, 2011; Belykh et al., 2013; Olenina, 2013). The phytoplankton composition there has been analyzed mostly microscopically. Brackish waters worldwide show diverse patterns, which were summarized for some lagoons by Lopes and Vasconcelos (2011). The dominance of picocyanobacterial species is reported for other lagoons, e.g., Patos Lagoon, Brazil or Florida Bay, United States (Phlips et al., 1999), Chesapeake Bay, United States (Wang and Chen, 2004; Chen et al., 2006) and Laguna Chascomús, Argentina (Iachetti and Llames, 2015) (**Table 1**). Similar phytoplankton communities are also found in freshwater lakes (e.g., Lake Santa Olalla, Spain (López-Archilla et al., 2004), Lake Baikal, Russia (Belykh et al., 2006), lakes in southern Sweden (Komárková-Legnerová and Cronberg, 1994), Guarapiranga Reservoir, Brazil (Santos et al., 2015), Vargem das Flores Reservoir, Brazil (Gomes et al., 2012), reservoirs along the Iguaçu River, Brazil (Silva et al., 2005) and in the Mount Cameroon region, Cameroon (Oben and Oben, 2006). Shallowness and a eutrophic state are common features of all these waters. Differences occur in colony-forming picocyanobacteria and APP assemblages, and in the temporal development. Most lagoons and lakes show enormous seasonal changes in the phytoplankton community. The DZBC has colony-forming picocyanobacteria and APP in high cell numbers and with high biovolume year-round, which makes it a special and unique system.

**Table 1:** Overview of articles on lagoons worldwide, with picocyanobacteria occurrence and dominance.

Lagoon^a^	Properties	Genera	Names according to	Method	Reference
Çaygören Reservoir		*Aphanocapsa, Aphanothece*	Komárek and Anagnostidis, 2005; names updated after AlgaeBase	Light microscopy	Çelık and Sevındık, 2015
Vistula Lagoon (POL)	shallow brackish eutrophic	*Aphanocapsa, Aphanothece, Cyanodictyon, Merismopedia, Snowella, Woronichinia*	Functional groups	Light microscopy, Lugol	Nawrocka and Kobos, 2011
Curonian Lagoon (LIT)	shallow brackish	some colonies	Not provided	Light microscopy, Lugol	Pilkaitytë, 2007
Venice Lagoon (ITA)	Near marine, shallow	*Coelosphaerium kutzingianum, C. minutissimum, Aphanothece, Synechococcus*	Not provided	Epifluorescence microscopy	Sorokin et al., 2004
Laguna de Tres Palos (MEX)	Oligohaline, hypereutrophic, shallow	*Aphanocapsa delicatissima, Merismopedia punctata, Chroococcus dispersus var. minor, Raphidiopsis curvata, Pseudanabaena limnetica*	Caljon, 1983; Moreno-Ruíz, 2000, 2005; Moreno, 2003	Light microscopy, Lugol	de la Lanza Espino et al., 2008
Patos Lagoon (BRA)	Shallow brackish	*Aphanocapsa nubilum, Planktolyngbya limnetica, Cyanodictyon imperfectum*	Not provided	Light microscopy	Torgan et al., 2006
Lake Santa Olalla (SPA)	Shallow hypertrophic	*Aphanothece, Chroococcus, Nostocales filaments*	Anagnostidis and Komárek, 1985; Waterbury, 1991	Epifluorescence microscopy, Formaldehyde	López-Archilla et al., 2004
Laguna Chascomús (ARG)	Fresh water shallow	*Aphanocapsa, Raphidiopsis, Planktolyngbya*	Not provided	Light microscopy, Lugol	Iachetti and Llames, 2015

*^a^Abbreviations for countries: ARG, Argentina; BRA, Brazil; ITA, Italy; LIT, Lithuania; MEX, Mexico; POL, Poland; SPA, Spain.*

### Features and Determination

The determination of picocyanobacteria is especially difficult due to their size and lack of microscopically identifiable features. The occurrence as single cells (APP) or a colony is the first distinctive feature. Single cells without a mucilage are determined as *Synechococcus*-like (rod-shaped) or *Synechocystis*-like (coccoid). Colony-forming genera are determined by the colony shape, presence or absence of a common mucilage, cell shape and size, or by special features such as gelatinous stalks. These are variable features, which may be absent or changed by environmental factors. However, there were some stable morphological features *in situ* which were used for determination of genera, e.g., gelatinous stalks of *Snowella*, general cell shape (rod-shaped or spherical) or aggregation in colonies (**Figure 2**) (Komárek and Anagnostidis, 1999, 2005; Komárek et al., 2014). According to Jasser and Callieri (2017b), the standard operation protocol is based on epi-fluorescence enumeration of picocyanobacteria. The determination of species by epifluorescence is not possible; instead, picocyanobacteria are devided by their main pigments [phycoerythrin (PE) or phycocyanine (PC) type].

The order Chroococcales (clade B2; e.g., the morphospecies *Aphanothece, Snowella, Woronichinia, Merismopedia*) shows a greater variety of stable features compared to the pico-size (0.2–2 μm) Synechococcales (clade C1), which consists mainly of very small, solitary, rod-shaped cells (Komárek and Anagnostidis, 1999; Komárek et al., 2014). The most abundant morpho-species was *Aphanothece.* No cultures could be established from this genus, perhaps because this genus was not present in the samples. Isolation of *Snowella* species was also not successful. Due to their distinctive characteristics, as there are presence of thin gelatinous stalks, radial and loose position of cells, the determination of *Snowella* by morphology seems to be correct and was verified by molecular genetic investigations (Rajaniemi-Wacklin et al., 2006). The identification of isolates that have been cultured for months or years can be difficult, due to the morphological changes and misleading molecular database information. Erroneous identifications in databases are caused mainly by misidentification of strains (Komárek and Anagnostidis, 1989) before sequencing and depositing the data under the wrong name. Further, specific literature on determinations is missing for marine, brackish and terrestrial habitats. Most species are determined in fixed samples, using the Süßwasserflora by Komárek and Anagnostidis (1989). The authors of these keys covered a wide range of cyanobacteria, but the focus in the Süßwasserflora is on freshwater habitats. Pankow (1990) described the phytoplankton of the brackish Baltic Sea region, and developed a key for determination, including drawings and micrographs, although only on morphological properties.

### Applied Morphological Aspects

Different researchers counted the APP, if it was recognized at all, in different ways. Some counted all pico-cyanobacteria as APP or *Synechococcus*-like (see Callieri, 2007 and references therein). We counted the free-living single cells as *Synechococcus* spp. The cells were rod-shaped and not part of a colony. Counting them as *Synechococcus* sp. was a pragmatic approach, because of the very few morphological features. Other investigations also used this categorization for analyses, e.g., in the flow cytometer (Schapira et al., 2010). The size, form, and absence of visible mucilage supported the morphological determination as *Synechococcus* sp. (Komárek and Anagnostidis, 1999). However, these cells could be fragments of colonies, when the colonies consisted of rod-shaped cells. Colonies with rod-shaped cells, such as *Aphanothece*, were very abundant in the DZBC (**Figure 2**), and there was no morphological feature indicating a separation between colony fragments and obligate solitary cells. Differentiation can only be assessed by molecular methods, to detect cryptic diversity. The molecular phylogeny cannot be reconstructed from the morphology (Palinska et al., 1996), especially for genera with very small cells. This leads to problems in determination up to order levels. The result is uncertainty about the ecophysiology and health risk potentials. Further, it leads to difficulties in the interpretation of monitoring results, because genera are set as indicator taxa (Reynolds et al., 2002; Padisák et al., 2009).

The classification in the Reynolds system (Reynolds et al., 2002) gave ambiguous results for the DZBC. The dominance of *Aphanothece* and *Aphanocapsa-*like morphospecies fits the shallow, turbid, nutrient-rich water of the DZBC. The result would be group K, according to Reynolds et al. (2002). A high pH tolerance was mentioned for this group as a special feature. Growth under light limitation and with changing salinities may be added as special features for the DZBC community. Group S1 (Reynolds et al., 2002) also matches the conditions found at the DZBC. The highly turbid, very low Secchi-depth waters (down to 20 cm) in the DZBC and the presence of *Pseudanabaena* and *Limnothrix* morphospecies concord with this group. Previous investigations in the DZBC found high abundances of *Synechococcus*. However, group Z can be excluded because it focuses mainly on oligotrophic marine waters. This classification into functional groups is based on microscopy counting data, and may serve as a rough estimation of the population features. These data are used by environmental agencies and are part of the Water Framework Directive (2000).

### Molecular Data

The combination of morphology and molecular data proved to be useful for the identification of the DZBC phytoplankton community. Morphological and molecular analyses *in situ* and *in vitro* gave partly contrasting results. These differences can be explained by culturing biases, misleading determination features, ambiguous database entries, and the plasticity of *Cyanobium* as shown by Jezberová and Komárková (2007a,b) and Huber et al. (2017). The focus was on the most conserved and reliable feature, the 16S rRNA gene. The 16S rRNA gene is used mostly to identify prokaryotes (Oren, 2011; Palinska and Surosz, 2014). The 16S rRNA gene is a conserved marker that reliably reveals the major phylogenetic lineages down to genus level (Woese, 1987). Phylogenies of 16S rRNA gene sequences showed that our isolates and many clone sequences were part of the *Cyanobium* clade. The affiliation to *Cyanobium* concords to the non-marine habitat conditions (Jasser and Callieri, 2017a).

The sequence data for the 16S rRNA gene clone support the occurrence of *Snowella in situ.* In contrast, no sequences of *Aphanothece* were found, although members of this genus were highly abundant according to morphological data. Many 16S rRNA gene sequences from the culture-independent cloning, as well as from the culture-dependent approach, instead belonged to the genus *Cyanobium*. The cell shape of *Cyanobium* is similar to that of *Aphanothece*. Major differences between these two genera are the formation of colonies and the position of the thylakoids. *Aphanothece* forms colonies and has irregular thylakoids. *Cyanobium* consists of solitary cells and has parietal thylakoids (Komárek et al., 2014). The position of the thylakoids cannot be determined with light microscopy, so the only remaining easily visible difference is the formation of colonies. For example, Jezberová and Komárková (2007a,b) and Huber et al. (2017) demonstrated the variability of this feature under different culture conditions. The formation of colonies depended on the presence of a grazer. Jezberová and Komárková (2007a,b) and Huber et al. (2017) conducted experiments using strains of the *Synechococcus–Prochlorococcus–Cyanobium* clade (SPC-clade), which were phylogenetically different *Cyanobium* lineages. These strains were determined as *Anathece* by Jezberová and Komárková (2007a), because of their ability to form colonies. The separation of *Anathece* from *Cyanobium* was based on morphology (Komárek et al., 2011), whereas there is no evidence for this in the phylogeny. Moreover, Huber et al. (2017) demonstrated the variability of this feature. The strains that we isolated belonged to the same *Cyanobium* lineages, but consisted of solitary cells under our grazer-free culture conditions. Grazers were present *in situ* (Heerkloss et al., 2006), so our isolates may form colonies under the stimulus of grazing, as shown for other morphological changes in green algae (Lampert et al., 1994). The small number of different genera in the DZBC is also reflected in a 16S rRNA gene DGGE fingerprint that resulted in only four bands (Schlie, 2009).

The 16S rRNA gene phylogeny (**Figure 8**) shows different lineages of the APP and colony-forming isolates. The clades consist of selected representatives of DZBC strains and closely related reference strains. It is possible that some of the DZBC isolates form a distinct clade (e.g., CZS 22C). Much more likely is that this gap will be filled by transitional strains. A bias in this respect is caused by the selection of reference strains included in this study. Only strains with a complete 16S rRNA gene sequence were included in the preliminary and the final analyses. Therefore, the well-studied Chesapeake Bay strains (Xu et al., 2015) and others were not included. The lack of comparable data sets is a major problem for genetic analyses. The same marker genes may be used (e.g., the ribosomal operon), but the sequence length (e.g., whole or partial 16S rRNA gene) and the exact marker position (e.g., 16S rRNA or ITS or 23S rRNA genes) vary in many cases. The number of known genome sequences has increased in recent years (e.g., Scanlan et al., 2009; Shih et al., 2013) and may eventually result in more comparable data sets.

### Conclusion and Importance for Applied Questions

Our investigations lead to two consequences: (i) Counted single cells are very likely genetically the same as the colonies, and (ii) the colonies found were members of *Cyanobium* (order Synechococcales/clade C1) and not *Aphanothece* (Chroococcales, clade B2). This hypothesis is supported by the fact that both our approaches showed no sequences of *Aphanothece*, even though this genus was highly abundant as determined morphologically. A sequence signal by the *Aphanothece*-like species (if present) could have been expected, because of the high cell numbers and the general cyanobacterial primers used.

The morphological features of APP and colony-forming picocyanobacteria are few, and depend strongly on the environmental conditions. Therefore, the determination of genera is difficult and misidentification often occurs. The genus *Aphanothece* seemed to be abundant in many eutrophic lagoons and lakes worldwide, as investigated morphologically. Molecular genetic investigations in waters with more or less similar environmental conditions gave different results. *Cyanobium* or *Synechococcus* sequences were often found, but not sequences of *Aphanothece* (**Table 2**). Phylogenetic analyses showed that many sequences determined as *Synechococcus* are actually *Cyanobium* (e.g., *Synechococcus* sp. WH5701). *Cyanobium* may be the most important pico-cyanobacterial (APP and colony-forming) contributor in eutrophic fresh and brackish waters worldwide.

**Table 2:** Occurrence of *Cyanobium* in brackish and fresh-water habitats, as inferred by genetic determinations.

Water system^a^	Reference	Method
Baltic Sea	Ernst et al., 2003; Haverkamp et al., 2009; Celepli et al., 2017	Culture isolation, 16S rRNA, metagenomes
Lake Constance (GER), Lake Maggiore (ITA), Lake Biwa (JAP)	Ernst et al., 2003	Culture isolation, 16S rRNA
Reservoirs Nová Říše, Římov, Vír (CZE)	Jezberová and Komárková, 2007b	Culture isolation, 16S rRNA
Lake Balaton (HU), Lake Zurich (SWI), Lake Constance (GER)	Ernst et al., 1999	Culture isolation, 16S rRNA
Albufera Lagoon (SPA)	Ghai et al., 2012	Metagenomes
Chesapeake Bay (USA)	Xu et al., 2015	Culture isolation, 16S rRNA
Coorong Lagoon (AUS)	Schapira et al., 2010	Flow cytometry

*^a^Abbreviations for countries: AUS, Australia; CZE, Czech Republic; GER, Germany; HU, Hungary; SWI, Switzerland; ITA, Italy; JAP, Japan; SPA, Spain; USA, United States of America*

Application of molecular genetic methods is necessary to identify genera and to validate microscopy data. The small size and variable morphology of pico-cyanobacteria make determination of members of this group through light microscopy uncertain and sometimes misleading. Linking of genetic species determination to morphology-based counting data should also be considered for the Reynolds functional-group classification. For example, addition of *Cyanobium* to the typical representatives of the K-group would allow molecular genetic data to be used as well. Calibration for such a revision could be done at stations where both morphospecies and molecular data are available. This would make research data more useful for environmental agencies, and provide a basis for the application of basic research data to monitoring. Problems still persist, because the nomenclature of cyanobacteria is not yet unified, even within the scientific community. They are either treated under the rules of Prokaryotes or under the former Botanical Nomenclature Code (Oren, 2011), but steps for unification are made (Oren and Garrity, 2014).

The dominant cyanobacteria species of the DZBC belong to the genus *Cyanobium.* It is very likely that in other lagoons where *Aphanothece* or *Synechococcus* have been reported in high numbers, *Cyanobium* is also the dominant phytoplankton genus, a suspicion that must be verified by molecular data. Therefore, the present view of the morphology of *Cyanobium* must be revised regarding colony and mucilage formation, as has begun with the work of Huber et al. (2017). The *in situ* morphology must be linked to the genetic marker, either by single-colony analysis or by fluorescence *in situ* hybridization (FisH) techniques. Comparison of *Cyanobium* sequences via qPCR against general cyanobacterial primers is another possible way to check the abundance of these picocyanobacteria in environmental samples. The application of next-generation 16S amplicon sequencing may also provide hints of the genetic diversity present in environmental samples, avoiding biases from culturing. The limitations of these methods are determined by the primer specificity and PCR biases. Nevertheless, they will give a deeper insight into the *in situ* genetic diversity than can be obtained with culture-based approaches.

## Author Contributions

MA: Introduction, Materials and Methods, Results, Discussion, except for the following contributions by the other authors. RS: Section Cell Counts (**Figures 2–4**), micrographs (**Figure 5**), section Applied Aspects in Discussion as well as conceptual basis. TP: Basis for alignment of sequences, conceptual work on aspects of molecular biology and molecular taxonomy.

## Conflict of Interest Statement

The authors declare that the research was conducted in the absence of any commercial or financial relationships that could be construed as a potential conflict of interest.

## Footnotes

1 www.algaebase.org

2 http://www.marinespecies.org
