# Supplementary material for: Identification of Cyanobacteria in a Eutrophic Coastal Lagoon on the Southern Baltic Coast
Source: Front Microbiol. 2017 May 29;8:923. doi: 10.3389/fmicb.2017.00923 (PMC5446986; doi:10.3389/fmicb.2017.00923)
Supplement: Supplementary file 1 [file Data_Sheet_1.docx]

Supplementary Material

**Identification of cyanobacteria in a eutrophic coastal lagoon on the southern Baltic coast**

**Martin Albrecht^*^, Thomas Pröschold, Rhena Schumann**

*** Correspondence:** Martin Albrecht [martin.albrecht@uni-rostock.de](mailto:martin.albrecht@uni-rostock.de)

**TABLES**

**Supplement table 1** Median of salinity, Secchi depth (cm), total phosphorus (TP, µmol l^-1^), total nitrogen to total phosphorus (TN:TP, µmol µmol^-1^) and Chlorophyll a (µg l^-1^) and pH ranges in 2006

|  | DB19 | DB16 | DB10 | ZS | DB06 | DB02 | DB01 |
| --- | --- | --- | --- | --- | --- | --- | --- |
| Salinity | 2.3 | 3.3 | 4.4 | 5.4 | 6.2 | 7.5 | 8.0 |
| pH | 8.1-9.4 | 8.1-9.4 | 8.2-9.5 | 7.8-9.2 | 8.1-9.1 | 8.0-9.3 | 8.0-9.2 |
| Secchi | 20 | 20 | 25 | 30 | 50 | 65 | 80 |
| TP | 2.6 | 1.8 | 1.5 | 1.5 | 1.4 | 1.1 | 0.8 |
| TN:TP | 77 | 124 | 127 | 106 | 103 | 98 | 110 |
| Chlorophyll a | 106 | 89 | 67 | 46 | 43 | 28 | 17 |
| n | 11 | 12 | 12 | 365, 50 for TP | 14, 13 for TP | | |

**Supplement table 2** Basionyms, synonyms and actual names of Baltic Sea/ DZBC genera. Phylogenetic position was retrieved from this works’ investigations. Isolate numbers refer to Rostock culture collection. * = includes clone sequences from Geiß *et al*., 2004 and this work.

| Name (Pankow 1990) | Synonyms/ Basionyms | Algaebase  (covered by Worms) | Newest name (phylogenetical tree) | Isolates (number) | Uncultured sequences* |
| --- | --- | --- | --- | --- | --- |
| *Aphanothece* |  | *?* | *Cyanobium* | 48 | 45 |
| *Cyanodictyon* |  | *?* | *Cyanobium* |  |  |
| *Cyanonephron* |  | *?* | *Cyanobium* |  |  |
| *Synechococcus* | *Anacystis* | *Synechococcus* | *Cyanobium* |  |  |
| *Lyngbya* | *Planktolyngbya* | *Planktolyngbya* | *-* | - | - |
| *Gomphosphaeria* | *Snowella* | *Snowella* | *Snowella* | - | 22 |
| *Oscillatoria* | *Limnothrix* | *Pseudanabaena* | *Pseudanabaena* | 7 | 4 |
| *Anabaenopsis* |  | *Cronbergia* | *Cronbergia* | 3 |  |

**Supplement table 3** Overview over phylogenetic clades according to Shih et al., 2013 (genome data), orders as published by Komárek et al., 2014, the assignment of the DZBC isolates and clones and the assignment of morphospecies according to type strain phylogenetic position

| Clades sensu Shih et al., 2013 (genome data) | Systematics after Komárek et al., 2014 (16S rRNA data) | Genera genetically found in the DZBC - isolates and clones (16S rRNA) | Morphospecies found in the DZBC |
| --- | --- | --- | --- |
| Clade A | Oscillatoriales |  |  |
| Clade B1 | Chroococcidiopsidales |  |  |
|  | Nostocales | *Cronbergia* | *Dolichospermum, Aphanizomenon, Cronbergia* |
| Clade B2 | Rubidibacter/Halothece |  |  |
|  | Chroococcales | *Snowella, Woronichinia* | *Snowella, Woronichinia, Aphanothece, Aphanocapsa, Cyanodictyon, Synechocystis* |
|  | Spirulinales |  |  |
| Clade B3 | Oscillatoriales |  |  |
| Clade C1 (α-Pico) | Synechococcales | *Cyanobium* |  |
| Clade C2 |  |  | *Synechococcus* |
| Clade C3 |  | *Leptolyngbya* |  |
| Clade D |  |  |  |
| Clade E |  |  |  |
| Clade F |  | *Pseudanabaena* | *Pseudanabaena, Limnothrix* |
| Clade G |  |  |  |
| *Gloeobacter violaceus* | Gloeobacterales |  |  |

**Supplement table 4** Isolate name, phylogenetic assignment to reference strain from fig. 9, sampling location, year of isolation and GenBank accession number.

| **Strain** | **Clade / displayed strain from fig. 9** | **Location** | **Month/ Year** | **Acc. number** |
| --- | --- | --- | --- | --- |
| CZS 25K | *Cyanobium* | Zingst Current | 05/2013 | KY379865 |
| CZS 24G | *Cyanobium* / CZS 24G | Zingst Current | 05/2013 | KY379862 |
| *CZS 24I* | *Cyanobium / CZS 24G* | *Zingst Current* | *05/2013* | *KY379863* |
| *CZS 48K* | *Cyanobium / CZS 24G* | *Zingst Current* | *05/2013* | *KY379889* |
| CZS 48M | *Cyanobium* / CZS 48M | Zingst Current | 05/2013 | KY379890 |
| *CZS 05I* | *Cyanobium / CZS 48M* | *Zingst Current* | *04/2013* | *KY379858* |
| *CZS 34I* | *Cyanobium / CZS 48M* | *Zingst Current* | *05/2013* | *KY379869* |
| *CZS 34J* | *Cyanobium / CZS 48M* | *Zingst Current* | *05/2013* | *KY379870* |
| *CZS 34K* | *Cyanobium / CZS 48M* | *Zingst Current* | *05/2013* | *KY379871* |
| *CZS 48D* | *Cyanobium / CZS 48M* | *Zingst Current* | *05/2013* | *KY379887* |
| *CZS 48J* | *Cyanobium / CZS 48M* | *Zingst Current* | *05/2013* | *KY379888* |
| CZS 25G | *Cyanobium* | Zingst Current | 05/2013 | KY379864 |
| CZS 27J | *Cyanobium* / CZS 27J | Zingst Current | 05/2013 | KY379866 |
| *CZS 3/1* | *Cyanobium / CZS 27J* | *Zingst Current* | *04/2009* | *KY379856* |
| *CZS 13D* | *Cyanobium / CZS 27J* | *Zingst Current* | *04/2013* | *KY379859* |
| *CZS 40E* | *Cyanobium / CZS 27J* | *Zingst Current* | *05/2013* | *KY379876* |
| *CZS 40F* | *Cyanobium / CZS 27J* | *Zingst Current* | *05/2013* | *KY379877* |
| CZS 34G | *Cyanobium* / CZS 34G | Zingst Current | 05/2013 | KY379868 |
| *CZS 3/4* | *Cyanobium / CZS 34G* | *Zingst Current* | *04/2009* | *KY379857* |

**Supplement table 4** continued

| **Strain** | **Clade / displayed strain from fig. 9** | **Location** | **Month/ Year** | **Acc. number** |
| --- | --- | --- | --- | --- |
| CZS 19G | *Cyanobium* / CZS 19G | Zingst Current | 04/2013 | KY379860 |
| *CZS 30E* | *Cyanobium / CZS 19G* | *Zingst Current* | *05/2013* | *KY379867* |
| *CZS 44D* | *Cyanobium / CZS 19G* | *Zingst Current* | *05/2013* | *KY379880* |
| *CZS 47C* | *Cyanobium / CZS 19G* | *Zingst Current* | *05/2013* | *KY379885* |
| CZS 40A | *Cyanobium* / CZS 40A | Zingst Current | 05/2013 | KY379875 |
| *CZS 39E* | *Cyanobium / CZS 40A* | *Zingst Current* | *05/2013* | *KY379874* |
| *CZS 41D* | *Cyanobium / CZS 40A* | *Zingst Current* | *05/2013* | *KY379878* |
| *CZS 43A* | *Cyanobium / CZS 40A* | *Zingst Current* | *05/2013* | *KY379879* |
| *CZS 46B* | *Cyanobium / CZS 40A* | *Zingst Current* | *05/2013* | *KY379884* |
| CZS 22C | *Cyanobium* | Zingst Current | 04/2013 | KY379861 |
| CZS 45I | *Cyanobium* | Zingst Current | 05/2013 | KY379883 |
| CZS 2/3 | *Merismopedia* | Zingst Current | 04/2009 | KY379855 |
| RK 2.2 | *Leptolyngbya* | Saaler Bodden | 09/2010 | KY379891 |
| *RK 2.3* | *Leptolyngbya* | *Saaler Bodden* | *09/2010* | *KY379892* |
| RK 4.3 | *Pseudanabaena* | Saaler Bodden | 09/2010 | KY379893 |
| *RK 4.6* | *Pseudanabaena* | *Saaler Bodden* | *09/2010* | *KY379894* |
| *CZS 35E* | *Pseudanabaena* | *Zingst Current* | *05/2013* | *KY379872* |
| *CZS 36B* | *Pseudanabaena* | *Zingst Current* | *05/2013* | *KY379873* |
| *CZS 45B* | *Pseudanabaena* | *Zingst Current* | *05/2013* | *KY379881* |
| *CZS 45C* | *Pseudanabaena* | *Zingst Current* | *05/2013* | *KY379882* |
| *CZS 47H* | *Pseudanabaena* | *Zingst Current* | *05/2013* | *KY379886* |
| SB 2.13 | *Cronbergia* | Saaler Bodden | 09/2010 | KY379895 |
| *SB 3.21* | *Cronbergia* | *Saaler Bodden* | *09/2010* | *KY379896* |
| *SB 3.23* | *Cronbergia* | *Saaler Bodden* | *09/2010* | *KY379897* |

**Supplement table 5** Clone sequences. BLAST results with first hit genus name. Phylogeny clade from 641 bp 16S rRNA sequences. Phylogenetic position by type strain: * = Synechococcales/ clade C1; ** = Chroococcales/ clade B2

| **Number of Clones** | **BLAST results** | **Phylogeny clade** |
| --- | --- | --- |
| 15 | *Synechococcus** | *Cyanobium** |
| 5 | *Cyanobacterium*** | *Cyanobium** |
| 5 | *Cyanobium** | *Cyanobium** |
| 3 | *Aphanocapsa*** | *Cyanobium** |
| 22 | *Snowella*** | *Snowella*** |
| 2 | *Limnococcus*** | *Gloeocapsa*** |
| 1 | *Woronichinia*** | *Woronichinia*** |

**Supplement table 6** 16S rRNA gene alignment as used for the phylogeny of the SPC-clade plus *Synechococcus elongatus* PCC6301 with base changes. Dots indicate the same bases as the uppermost sequence. The letters indicate base changes. Cya=*Cyanobium*, mar_Syn= marine *Synechococcus*, Pro_mar=*Prochlorococcus marinus*, S_elong=*Synechococcus elongatus*.

10 20 30 40 50

....|....|....|....|....|....|....|....|....|....|

**Cya_CZS_24G_KY379862**  **AUGAACGCUGGCGGCGUGCUUAACACAUGCAAGUCGAACGAGUGGCGGAC**

**'Syn'_WH_5701_AY172832**  **..................................................**

**Cya_CZS_25K_KY379865**  **........G.........................................**

**Cya_CZS_48M_KY379890**  **..U...............................................**

**Cya_SAG_3_81_NR125481**  **..................................................**

**Cya_CZS_45I_KY379883**  **..................................................**

**Cya_Suigetsu_CR1**  **..................................................**

**Cya_PCC_7001_AM709626**  **..................................................**

**Cya_CZS_34G_KY379868**  **..................................................**

**Cya_CZS_27J_KY379866**  **..................................................**

**Cya_BS4_AF330252**  **..................................................**

**Cya_CZS_25G_KY379864**  **..................................................**

**Cya_CZS_22C_KY379861**  **..................................................**

**Cya_CZS_19G_KY379860**  **..................................................**

**Cya_CZS_40A_KY379875**  **..................................................**

**Cya_gra_PCC_6307_NR114406** **..................................................**

**Cya_Suigetsu_CG1_AB610890** **..................................................**

**mar_Syn_WH_7803_AF081834**  **..................................................**

**Pro_mar_MIT_9301_NC009091** **..................................................**

**S_elong_PCC_6301_NR074309** **..................................................**

60 70 80 90 100

....|....|....|....|....|....|....|....|....|....|

**Cya_CZS_24G_KY379862**  **GGGUGAGUAACGCGUGGGAAUCUGCCCUCAGGAGGGGGAUAACGGCCGGA**

**'Syn'_WH_5701_AY172832**  **..................................................**

**Cya_CZS_25K_KY379865**  **..................................................**

**Cya_CZS_48M_KY379890**  **..................................................**

**Cya_SAG_3_81_NR125481**  **................A.............................U...**

**Cya_CZS_45I_KY379883**  **................A.............................U...**

**Cya_Suigetsu_CR1**  **................A.............................U...**

**Cya_PCC_7001_AM709626**  **................A..........CA.................U...**

**Cya_CZS_34G_KY379868**  **................A.............................U...**

**Cya_CZS_27J_KY379866**  **................A.............................U...**

**Cya_BS4_AF330252**  **................A.............................U...**

**Cya_CZS_25G_KY379864**  **................A.............................U...**

**Cya_CZS_22C_KY379861**  **................A.............................U...**

**Cya_CZS_19G_KY379860**  **.............................................UU...**

**Cya_CZS_40A_KY379875**  **..............................................U...**

**Cya_gra_PCC_6307_NR114406** **..................................................**

**Cya_Suigetsu_CG1_AB610890** **..................................................**

**mar_Syn_WH_7803_AF081834**  **..............................................U...**

**Pro_mar_MIT_9301_NC009091** **................A............................UU...**

**S_elong_PCC_6301_NR074309** **................A.........UA.....C.....C...A.UU...**

110 120 130 140 150

....|....|....|....|....|....|....|....|....|....|

**Cya_CZS_24G_KY379862**  **AACGGCCGCUAAUACCCCAUAUGCCGAGAGGUGAAACGAAUUUCGCCUGG**

**'Syn'_WH_5701_AY172832**  **..................................................**

**Cya_CZS_25K_KY379865**  **..................................................**

**Cya_CZS_48M_KY379890**  **.................................................A**

**Cya_SAG_3_81_NR125481**  **....................................U............A**

**Cya_CZS_45I_KY379883**  **....................................U............A**

**Cya_Suigetsu_CR1**  **..........................CA........U...A........A**

**Cya_PCC_7001_AM709626**  **.......................................GA.......U.**

**Cya_CZS_34G_KY379868**  **.......................................G.........A**

**Cya_CZS_27J_KY379866**  **....................................U............A**

**Cya_BS4_AF330252**  **....................................U............A**

**Cya_CZS_25G_KY379864**  **....................................U............A**

**Cya_CZS_22C_KY379861**  **....................................U............A**

**Cya_CZS_19G_KY379860**  **....A...............................U............A**

**Cya_CZS_40A_KY379875**  **....................................U............A**

**Cya_gra_PCC_6307_NR114406** **....................................U............A**

**Cya_Suigetsu_CG1_AB610890** **....................................U............A**

**mar_Syn_WH_7803_AF081834**  **....................................U............A**

**Pro_mar_MIT_9301_NC009091** **....A....................U.CU.......U............A**

**S_elong_PCC_6301_NR074309** **....A.U..........G..G................AUU.A.G.....U**

160 170 180 190 200

....|....|....|....|....|....|....|....|....|....|

**Cya_CZS_24G_KY379862**  **GGAUGAGCCCGCGUCUGAUUAGCUAGUUGGUGAGGUAAUGGCUCACCAAG**

**'Syn'_WH_5701_AY172832**  **..................................................**

**Cya_CZS_25K_KY379865**  **..................................................**

**Cya_CZS_48M_KY379890**  **..................................................**

**Cya_SAG_3_81_NR125481**  **........U.......................U.....A...........**

**Cya_CZS_45I_KY379883**  **........U.......................U.....A...........**

**Cya_Suigetsu_CR1**  **........U.......................G.....GA..CU......**

**Cya_PCC_7001_AM709626**  **........U.......................G.....GA..CU......**

**Cya_CZS_34G_KY379868**  **........U..............................A..........**

**Cya_CZS_27J_KY379866**  **........U.......................G.....GA..CU......**

**Cya_BS4_AF330252**  **........U.......................G.....GA..CU......**

**Cya_CZS_25G_KY379864**  **........U.......................G.....GUA.C.UA.C.A**

**Cya_CZS_22C_KY379861**  **........U.......................G.........C.......**

**Cya_CZS_19G_KY379860**  **................................G.........C.......**

**Cya_CZS_40A_KY379875**  **................................G.........C.......**

**Cya_gra_PCC_6307_NR114406** **................................G.........C.......**

**Cya_Suigetsu_CG1_AB610890** **................................G.........C.......**

**mar_Syn_WH_7803_AF081834**  **......................................GA..........**

**Pro_mar_MIT_9301_NC009091** **........U.........................................**

**S_elong_PCC_6301_NR074309** **A.......U.......................G.....G...CU......**

210 220 230 240 250

....|....|....|....|....|....|....|....|....|....|

**Cya_CZS_24G_KY379862**  **GCUUCGAUCAGUAGCUGGUCUGAGAGGAUGAUCAGCCACACUGGGACUGA**

**'Syn'_WH_5701_AY172832**  **..A...............................................**

**Cya_CZS_25K_KY379865**  **.............U....................C...............**

**Cya_CZS_48M_KY379890**  **..A...............................................**

**Cya_SAG_3_81_NR125481**  **..................................................**

**Cya_CZS_45I_KY379883**  **..................................................**

**Cya_Suigetsu_CR1**  **..A...............................................**

**Cya_PCC_7001_AM709626**  **..A...............................................**

**Cya_CZS_34G_KY379868**  **..A...............................................**

**Cya_CZS_27J_KY379866**  **..................................................**

**Cya_BS4_AF330252**  **..................................................**

**Cya_CZS_25G_KY379864**  **.GC...............................................**

**Cya_CZS_22C_KY379861**  **..................................................**

**Cya_CZS_19G_KY379860**  **..GA..............................................**

**Cya_CZS_40A_KY379875**  **..GA..............................................**

**Cya_gra_PCC_6307_NR114406** **..GA..............................................**

**Cya_Suigetsu_CG1_AB610890** **..GA..............................................**

**mar_Syn_WH_7803_AF081834**  **..A...............................................**

**Pro_mar_MIT_9301_NC009091** **..................................................**

**S_elong_PCC_6301_NR074309** **..GA..............................................**

260 270 280 290 300

....|....|....|....|....|....|....|....|....|....|

**Cya_CZS_24G_KY379862**  **GACACGGCCCAGACUCCUACGGGAGGCAGCAGUGGGGAAUUUUCCGCAAU**

**'Syn'_WH_5701_AY172832**  **..................................................**

**Cya_CZS_25K_KY379865**  **..................................................**

**Cya_CZS_48M_KY379890**  **..................................................**

**Cya_SAG_3_81_NR125481**  **..................................................**

**Cya_CZS_45I_KY379883**  **..................................................**

**Cya_Suigetsu_CR1**  **..................................................**

**Cya_PCC_7001_AM709626**  **..................................................**

**Cya_CZS_34G_KY379868**  **..................................................**

**Cya_CZS_27J_KY379866**  **..................................................**

**Cya_BS4_AF330252**  **..................................................**

**Cya_CZS_25G_KY379864**  **..................................................**

**Cya_CZS_22C_KY379861**  **..................................................**

**Cya_CZS_19G_KY379860**  **..................................................**

**Cya_CZS_40A_KY379875**  **..................................................**

**Cya_gra_PCC_6307_NR114406** **..................................................**

**Cya_Suigetsu_CG1_AB610890** **..................................................**

**mar_Syn_WH_7803_AF081834**  **..................................................**

**Pro_mar_MIT_9301_NC009091** **..................................................**

**S_elong_PCC_6301_NR074309** **..................................................**

310 320 330 340 350

....|....|....|....|....|....|....|....|....|....|

**Cya_CZS_24G_KY379862**  **GGGCGCAAGCCUGACGGAGCAACGCCGCGUGAGGGACGAAGGCCUCUGGG**

**'Syn'_WH_5701_AY172832**  **..................................................**

**Cya_CZS_25K_KY379865**  **...A..U...AA..A.....................U.............**

**Cya_CZS_48M_KY379890**  **..................................................**

**Cya_SAG_3_81_NR125481**  **....................................U.............**

**Cya_CZS_45I_KY379883**  **....................................U.............**

**Cya_Suigetsu_CR1**  **.....A..............................U.............**

**Cya_PCC_7001_AM709626**  **.....A..............................U.............**

**Cya_CZS_34G_KY379868**  **.....A..............................U.............**

**Cya_CZS_27J_KY379866**  **.....A..............................U.............**

**Cya_BS4_AF330252**  **.....A..............................U.............**

**Cya_CZS_25G_KY379864**  **.....A..............................U.............**

**Cya_CZS_22C_KY379861**  **.....A..............................U.............**

**Cya_CZS_19G_KY379860**  **.....A..............................U.............**

**Cya_CZS_40A_KY379875**  **.....A..............................U.............**

**Cya_gra_PCC_6307_NR114406** **.....A..............................U.............**

**Cya_Suigetsu_CG1_AB610890** **.....A..............................U.............**

**mar_Syn_WH_7803_AF081834**  **.....A..............................U.............**

**Pro_mar_MIT_9301_NC009091** **.....A............................................**

**S_elong_PCC_6301_NR074309** **...............................G....G.....UU.U...A**

360 370 380 390 400

....|....|....|....|....|....|....|....|....|....|

**Cya_CZS_24G_KY379862**  **CUGUAAACCUCUUUUCUCAAGGAAGAAGAAUGACGGUACUUGAGGAAUAA**

**'Syn'_WH_5701_AY172832**  **..................................................**

**Cya_CZS_25K_KY379865**  **..................................................**

**Cya_CZS_48M_KY379890**  **..................................................**

**Cya_SAG_3_81_NR125481**  **...............A.............C.............U......**

**Cya_CZS_45I_KY379883**  **...............A.............C.............U......**

**Cya_Suigetsu_CR1**  **...............A.............C.............U......**

**Cya_PCC_7001_AM709626**  **.............................C....................**

**Cya_CZS_34G_KY379868**  **.............................C....................**

**Cya_CZS_27J_KY379866**  **.............................C....................**

**Cya_BS4_AF330252**  **.............................C....................**

**Cya_CZS_25G_KY379864**  **..................................................**

**Cya_CZS_22C_KY379861**  **..................................................**

**Cya_CZS_19G_KY379860**  **..................................................**

**Cya_CZS_40A_KY379875**  **..................................................**

**Cya_gra_PCC_6307_NR114406** **..................................................**

**Cya_Suigetsu_CG1_AB610890** **..................................................**

**mar_Syn_WH_7803_AF081834**  **.............................C....................**

**Pro_mar_MIT_9301_NC009091** **..................................................**

**S_elong_PCC_6301_NR074309** **.........C.........G.........G.........C..........**

410 420 430 440 450

....|....|....|....|....|....|....|....|....|....|

**Cya_CZS_24G_KY379862**  **GCCACGGCUAAUUCCGUGCCAGCAGCCGCGGUAAUACGGGAGUGGCAAGC**

**'Syn'_WH_5701_AY172832**  **..................................................**

**Cya_CZS_25K_KY379865**  **..................................................**

**Cya_CZS_48M_KY379890**  **..................................................**

**Cya_SAG_3_81_NR125481**  **..................................................**

**Cya_CZS_45I_KY379883**  **..................................................**

**Cya_Suigetsu_CR1**  **..................................................**

**Cya_PCC_7001_AM709626**  **..................................................**

**Cya_CZS_34G_KY379868**  **..................................................**

**Cya_CZS_27J_KY379866**  **..................................................**

**Cya_BS4_AF330252**  **..................................................**

**Cya_CZS_25G_KY379864**  **..................................................**

**Cya_CZS_22C_KY379861**  **..................................................**

**Cya_CZS_19G_KY379860**  **..................................................**

**Cya_CZS_40A_KY379875**  **..................................................**

**Cya_gra_PCC_6307_NR114406** **..................................................**

**Cya_Suigetsu_CG1_AB610890** **..................................................**

**mar_Syn_WH_7803_AF081834**  **..................................................**

**Pro_mar_MIT_9301_NC009091** **..................................................**

**S_elong_PCC_6301_NR074309** **...U......................................A.......**

460 470 480 490 500

....|....|....|....|....|....|....|....|....|....|

**Cya_CZS_24G_KY379862**  **GUUAUCCGGAAUUAUUGGGCGUAAAGCGUCCGCAGGCGGCCUUGAAAGUC**

**'Syn'_WH_5701_AY172832**  **..................................................**

**Cya_CZS_25K_KY379865**  **..................................................**

**Cya_CZS_48M_KY379890**  **..................................................**

**Cya_SAG_3_81_NR125481**  **.......................................UU..AC.....**

**Cya_CZS_45I_KY379883**  **.......................................UU..AC.....**

**Cya_Suigetsu_CR1**  **.......................................UU...U.....**

**Cya_PCC_7001_AM709626**  **..................................................**

**Cya_CZS_34G_KY379868**  **...........................................UU.....**

**Cya_CZS_27J_KY379866**  **..................................................**

**Cya_BS4_AF330252**  **..................................................**

**Cya_CZS_25G_KY379864**  **..................................................**

**Cya_CZS_22C_KY379861**  **..................................................**

**Cya_CZS_19G_KY379860**  **............................................U.....**

**Cya_CZS_40A_KY379875**  **............................................U.....**

**Cya_gra_PCC_6307_NR114406** **............................................U.....**

**Cya_Suigetsu_CG1_AB610890** **............................................U.....**

**mar_Syn_WH_7803_AF081834**  **.........................................C.UC.....**

**Pro_mar_MIT_9301_NC009091** **........................................U..UC.....**

**S_elong_PCC_6301_NR074309** **.......................................UUAAUC.....**

510 520 530 540 550

....|....|....|....|....|....|....|....|....|....|

**Cya_CZS_24G_KY379862**  **UGUUGUUAAAGCGUGGAGCUCAACUCCAUUUAAGCAAUGGAAACUACAAG**

**'Syn'_WH_5701_AY172832**  **...............................C..................**

**Cya_CZS_25K_KY379865**  **..................................................**

**Cya_CZS_48M_KY379890**  **...............................C..................**

**Cya_SAG_3_81_NR125481**  **...C......A....................CG..G.........GU...**

**Cya_CZS_45I_KY379883**  **...C...........................CG..G.........GU...**

**Cya_Suigetsu_CR1**  **.............................................G....**

**Cya_PCC_7001_AM709626**  **....................U..........C..............G...**

**Cya_CZS_34G_KY379868**  **......C.............U..........CG............GGG..**

**Cya_CZS_27J_KY379866**  **..C.................U..........C....G.............**

**Cya_BS4_AF330252**  **..C.................U..........C....G.............**

**Cya_CZS_25G_KY379864**  **..C.................U..........C...GG.............**

**Cya_CZS_22C_KY379861**  **....................U..........C...G..............**

**Cya_CZS_19G_KY379860**  **...C................U..........C...G..............**

**Cya_CZS_40A_KY379875**  **...C................U..........C...G..............**

**Cya_gra_PCC_6307_NR114406** **...C................U..........C...G..............**

**Cya_Suigetsu_CG1_AB610890** **...C................U..............G..............**

**mar_Syn_WH_7803_AF081834**  **..C.......AA........U........CAUG...G........GUUG.**

**Pro_mar_MIT_9301_NC009091** **..C.................U........CAUG...G........GA...**

**S_elong_PCC_6301_NR074309** **......C.........G.......CU...AC.G............GAUU.**

560 570 580 590 600

....|....|....|....|....|....|....|....|....|....|

**Cya_CZS_24G_KY379862**  **GCUAGAGUGUGGUAGGGGCAGAGGGAAUUCCCGGUGUAGCGGUGAAAUGC**

**'Syn'_WH_5701_AY172832**  **..................................................**

**Cya_CZS_25K_KY379865**  **..................................................**

**Cya_CZS_48M_KY379890**  **..................................................**

**Cya_SAG_3_81_NR125481**  **A.................................................**

**Cya_CZS_45I_KY379883**  **A.................................................**

**Cya_Suigetsu_CR1**  **A.................................................**

**Cya_PCC_7001_AM709626**  **..................................................**

**Cya_CZS_34G_KY379868**  **...U..............................................**

**Cya_CZS_27J_KY379866**  **..................................................**

**Cya_BS4_AF330252**  **..................................................**

**Cya_CZS_25G_KY379864**  **..................................................**

**Cya_CZS_22C_KY379861**  **..................................................**

**Cya_CZS_19G_KY379860**  **...U..............................................**

**Cya_CZS_40A_KY379875**  **...U..............................................**

**Cya_gra_PCC_6307_NR114406** **...U..............................................**

**Cya_Suigetsu_CG1_AB610890** **...U..............................................**

**mar_Syn_WH_7803_AF081834**  **...U..............................................**

**Pro_mar_MIT_9301_NC009091** **...U....A.........................................**

**S_elong_PCC_6301_NR074309** **A.......A.........U..C.........A..................**

610 620 630 640 650

....|....|....|....|....|....|....|....|....|....|

**Cya_CZS_24G_KY379862**  **GUAGAUAUCGGGAAGAACACCAGUGGCGAAGGCGCUCUGCUGGGCCAUAA**

**'Syn'_WH_5701_AY172832**  **..................................................**

**Cya_CZS_25K_KY379865**  **..................................................**

**Cya_CZS_48M_KY379890**  **..................................................**

**Cya_SAG_3_81_NR125481**  **..................................................**

**Cya_CZS_45I_KY379883**  **..................................................**

**Cya_Suigetsu_CR1**  **..................................................**

**Cya_PCC_7001_AM709626**  **..................................................**

**Cya_CZS_34G_KY379868**  **..................................................**

**Cya_CZS_27J_KY379866**  **..................................................**

**Cya_BS4_AF330252**  **...N..............................................**

**Cya_CZS_25G_KY379864**  **..................................................**

**Cya_CZS_22C_KY379861**  **..................................................**

**Cya_CZS_19G_KY379860**  **..................................................**

**Cya_CZS_40A_KY379875**  **..................................................**

**Cya_gra_PCC_6307_NR114406** **..................................................**

**Cya_Suigetsu_CG1_AB610890** **..................................................**

**mar_Syn_WH_7803_AF081834**  **..................................................**

**Pro_mar_MIT_9301_NC009091** **................................................U.**

**S_elong_PCC_6301_NR074309** **.........U.............C......A....G..A...........**

660 670 680 690 700

....|....|....|....|....|....|....|....|....|....|

**Cya_CZS_24G_KY379862**  **CUGACGCUCAUGGACGAAAGCCAGGGGAGCGAAAGGGAUUAGAUACCCCU**

**'Syn'_WH_5701_AY172832**  **..................................................**

**Cya_CZS_25K_KY379865**  **..................................................**

**Cya_CZS_48M_KY379890**  **..................................................**

**Cya_SAG_3_81_NR125481**  **..................................................**

**Cya_CZS_45I_KY379883**  **..................................................**

**Cya_Suigetsu_CR1**  **..................................................**

**Cya_PCC_7001_AM709626**  **..................................................**

**Cya_CZS_34G_KY379868**  **..................................................**

**Cya_CZS_27J_KY379866**  **..................................................**

**Cya_BS4_AF330252**  **.........................................N..N.....**

**Cya_CZS_25G_KY379864**  **..................................................**

**Cya_CZS_22C_KY379861**  **..................................................**

**Cya_CZS_19G_KY379860**  **..................................................**

**Cya_CZS_40A_KY379875**  **..................................................**

**Cya_gra_PCC_6307_NR114406** **..................................................**

**Cya_Suigetsu_CG1_AB610890** **..................................................**

**mar_Syn_WH_7803_AF081834**  **..................................................**

**Pro_mar_MIT_9301_NC009091** **..................................................**

**S_elong_PCC_6301_NR074309** **.....................U............................**

710 720 730 740 750

....|....|....|....|....|....|....|....|....|....|

**Cya_CZS_24G_KY379862**  **GUAGUCCUGGCCGUAAACGAUGAACACUAGGUGUCGGGAGAAUUAACCCU**

**'Syn'_WH_5701_AY172832**  **..................................................**

**Cya_CZS_25K_KY379865**  **..................................................**

**Cya_CZS_48M_KY379890**  **..................................................**

**Cya_SAG_3_81_NR125481**  **......................................G....CG....C**

**Cya_CZS_45I_KY379883**  **......................................G....CG....C**

**Cya_Suigetsu_CR1**  **......................................G....CG....C**

**Cya_PCC_7001_AM709626**  **......................................G....CG....C**

**Cya_CZS_34G_KY379868**  **......................................G....CG....C**

**Cya_CZS_27J_KY379866**  **......................................G....CG....C**

**Cya_BS4_AF330252**  **......................................G....CG....C**

**Cya_CZS_25G_KY379864**  **......................................G....CG....C**

**Cya_CZS_22C_KY379861**  **......................................G....CG....C**

**Cya_CZS_19G_KY379860**  **......................................G....CG....C**

**Cya_CZS_40A_KY379875**  **......................................G....CG....C**

**Cya_gra_PCC_6307_NR114406** **......................................G....CG....C**

**Cya_Suigetsu_CG1_AB610890** **......................................G....CG....C**

**mar_Syn_WH_7803_AF081834**  **......................................G....CG....C**

**Pro_mar_MIT_9301_NC009091** **......................................G....CG....C**

**S_elong_PCC_6301_NR074309** **........A.........................U.C.U....CG....G**

760 770 780 790 800

....|....|....|....|....|....|....|....|....|....|

**Cya_CZS_24G_KY379862**  **UCCGGUGUCGUAGCCAACGCGUUAAGUGUUCCGCCUGGGGAGUACGCACG**

**'Syn'_WH_5701_AY172832**  **..................................................**

**Cya_CZS_25K_KY379865**  **.............................................U....**

**Cya_CZS_48M_KY379890**  **.U................................................**

**Cya_SAG_3_81_NR125481**  **CU................................................**

**Cya_CZS_45I_KY379883**  **CU................................................**

**Cya_Suigetsu_CR1**  **CU................................................**

**Cya_PCC_7001_AM709626**  **CU................................................**

**Cya_CZS_34G_KY379868**  **CU................................................**

**Cya_CZS_27J_KY379866**  **..................................................**

**Cya_BS4_AF330252**  **CU................................................**

**Cya_CZS_25G_KY379864**  **..............U...................................**

**Cya_CZS_22C_KY379861**  **CU................................................**

**Cya_CZS_19G_KY379860**  **CU............U...................................**

**Cya_CZS_40A_KY379875**  **CU............U...................................**

**Cya_gra_PCC_6307_NR114406** **CU............U...................................**

**Cya_Suigetsu_CG1_AB610890** **CU............U...................................**

**mar_Syn_WH_7803_AF081834**  **CU................................................**

**Pro_mar_MIT_9301_NC009091** **.U............U...................................**

**S_elong_PCC_6301_NR074309** **CG.A...C..........................................**

810 820 830 840 850

....|....|....|....|....|....|....|....|....|....|

**Cya_CZS_24G_KY379862**  **CAAGUGUGAAACUCAAAGGAAUUGACGGGGGCCCGCACAAGCGGUGGAGU**

**'Syn'_WH_5701_AY172832**  **..................................................**

**Cya_CZS_25K_KY379865**  **.................A................................**

**Cya_CZS_48M_KY379890**  **..................................................**

**Cya_SAG_3_81_NR125481**  **..................................................**

**Cya_CZS_45I_KY379883**  **..................................................**

**Cya_Suigetsu_CR1**  **..................................................**

**Cya_PCC_7001_AM709626**  **..................................................**

**Cya_CZS_34G_KY379868**  **..................................................**

**Cya_CZS_27J_KY379866**  **..................................................**

**Cya_BS4_AF330252**  **..................................................**

**Cya_CZS_25G_KY379864**  **..................................................**

**Cya_CZS_22C_KY379861**  **..................................................**

**Cya_CZS_19G_KY379860**  **..................................................**

**Cya_CZS_40A_KY379875**  **..................................................**

**Cya_gra_PCC_6307_NR114406** **..................................................**

**Cya_Suigetsu_CG1_AB610890** **..................................................**

**mar_Syn_WH_7803_AF081834**  **..................................................**

**Pro_mar_MIT_9301_NC009091** **..................................................**

**S_elong_PCC_6301_NR074309** **..................................................**

860 870 880 890 900

....|....|....|....|....|....|....|....|....|....|

**Cya_CZS_24G_KY379862**  **AUGUGGUUUAAUUCGAUGCAACGCGAAGAACCUUACCAGGGUUUGACAUC**

**'Syn'_WH_5701_AY172832**  **..................................................**

**Cya_CZS_25K_KY379865**  **..................................................**

**Cya_CZS_48M_KY379890**  **..................................................**

**Cya_SAG_3_81_NR125481**  **..................................................**

**Cya_CZS_45I_KY379883**  **..................................................**

**Cya_Suigetsu_CR1**  **..................................................**

**Cya_PCC_7001_AM709626**  **..................................................**

**Cya_CZS_34G_KY379868**  **..................................................**

**Cya_CZS_27J_KY379866**  **..................................................**

**Cya_BS4_AF330252**  **..................................................**

**Cya_CZS_25G_KY379864**  **..................................................**

**Cya_CZS_22C_KY379861**  **..................................................**

**Cya_CZS_19G_KY379860**  **..................................................**

**Cya_CZS_40A_KY379875**  **..................................................**

**Cya_gra_PCC_6307_NR114406** **..................................................**

**Cya_Suigetsu_CG1_AB610890** **..................................................**

**mar_Syn_WH_7803_AF081834**  **..................................................**

**Pro_mar_MIT_9301_NC009091** **..................................................**

**S_elong_PCC_6301_NR074309** **..................................................**

910 920 930 940 950

....|....|....|....|....|....|....|....|....|....|

**Cya_CZS_24G_KY379862**  **CUGCGAAUCCCUUGGAAACGAGGGAGUGCCUUCGGGAACGCAGUGACAGG**

**'Syn'_WH_5701_AY172832**  **...................UU.............................**

**Cya_CZS_25K_KY379865**  **.....................................G.....A......**

**Cya_CZS_48M_KY379890**  **..........U........UUA...............G............**

**Cya_SAG_3_81_NR125481**  **........U..........UU..A.............G.....A......**

**Cya_CZS_45I_KY379883**  **........U..........UU..A.............G............**

**Cya_Suigetsu_CR1**  **.........U............A...........................**

**Cya_PCC_7001_AM709626**  **...................UU................G.....A......**

**Cya_CZS_34G_KY379868**  **...................UU................G.....A......**

**Cya_CZS_27J_KY379866**  **.....................................G............**

**Cya_BS4_AF330252**  **.....................................G............**

**Cya_CZS_25G_KY379864**  **.....................................G.....A......**

**Cya_CZS_22C_KY379861**  **.....................................G............**

**Cya_CZS_19G_KY379860**  **.....................................G.....A......**

**Cya_CZS_40A_KY379875**  **.....................................G.....A......**

**Cya_gra_PCC_6307_NR114406** **.....................................G.....A......**

**Cya_Suigetsu_CG1_AB610890** **.....................................G.....A......**

**mar_Syn_WH_7803_AF081834**  **.......C.U............A.G.........................**

**Pro_mar_MIT_9301_NC009091** **.......C.U...A....UUU.A.G.............U...........**

**S_elong_PCC_6301_NR074309** **.CC......U............A..............G..GG.A......**

960 970 980 990 1000

....|....|....|....|....|....|....|....|....|....|

**Cya_CZS_24G_KY379862**  **UGGUGCAUGGCUGUCGUCAGCUCGUGUCGUGAGAUGUUGGGUUAAGUCCC**

**'Syn'_WH_5701_AY172832**  **..................................................**

**Cya_CZS_25K_KY379865**  **..................................................**

**Cya_CZS_48M_KY379890**  **..................................................**

**Cya_SAG_3_81_NR125481**  **..................................................**

**Cya_CZS_45I_KY379883**  **..................................................**

**Cya_Suigetsu_CR1**  **..................................................**

**Cya_PCC_7001_AM709626**  **..................................................**

**Cya_CZS_34G_KY379868**  **..................................................**

**Cya_CZS_27J_KY379866**  **..................................................**

**Cya_BS4_AF330252**  **..................................................**

**Cya_CZS_25G_KY379864**  **..................................................**

**Cya_CZS_22C_KY379861**  **..................................................**

**Cya_CZS_19G_KY379860**  **..................................................**

**Cya_CZS_40A_KY379875**  **..................................................**

**Cya_gra_PCC_6307_NR114406** **..................................................**

**Cya_Suigetsu_CG1_AB610890** **..................................................**

**mar_Syn_WH_7803_AF081834**  **..................................................**

**Pro_mar_MIT_9301_NC009091** **..................................................**

**S_elong_PCC_6301_NR074309** **..................................................**

1010 1020 1030 1040 1050

....|....|....|....|....|....|....|....|....|....|

**Cya_CZS_24G_KY379862**  **GCAACGAGCGCAACCCACGUCUUUAGUUGCCAGCAUUCAGUUGGGCACUC**

**'Syn'_WH_5701_AY172832**  **..................................................**

**Cya_CZS_25K_KY379865**  **.....................................G............**

**Cya_CZS_48M_KY379890**  **.....................................G............**

**Cya_SAG_3_81_NR125481**  **.....................................A............**

**Cya_CZS_45I_KY379883**  **.....................................U............**

**Cya_Suigetsu_CR1**  **.....................G...............U............**

**Cya_PCC_7001_AM709626**  **.....................................G............**

**Cya_CZS_34G_KY379868**  **.....................................A............**

**Cya_CZS_27J_KY379866**  **.....................................G............**

**Cya_BS4_AF330252**  **.....................................G............**

**Cya_CZS_25G_KY379864**  **.....................................G............**

**Cya_CZS_22C_KY379861**  **..................................................**

**Cya_CZS_19G_KY379860**  **..................................................**

**Cya_CZS_40A_KY379875**  **..................................................**

**Cya_gra_PCC_6307_NR114406** **..................................................**

**Cya_Suigetsu_CG1_AB610890** **..................................................**

**mar_Syn_WH_7803_AF081834**  **.....................................U............**

**Pro_mar_MIT_9301_NC009091** **....................U................U............**

**S_elong_PCC_6301_NR074309** **....................U...........U.................**

1060 1070 1080 1090 1100

....|....|....|....|....|....|....|....|....|....|

**Cya_CZS_24G_KY379862**  **UAGAGAGACCGCCGGUGAUAAACCGGAGGAAGGUGUGGAUGACGUCAAGU**

**'Syn'_WH_5701_AY172832**  **..................................................**

**Cya_CZS_25K_KY379865**  **..................................................**

**Cya_CZS_48M_KY379890**  **..................................................**

**Cya_SAG_3_81_NR125481**  **..................................................**

**Cya_CZS_45I_KY379883**  **..................................................**

**Cya_Suigetsu_CR1**  **...C..............................................**

**Cya_PCC_7001_AM709626**  **..................................................**

**Cya_CZS_34G_KY379868**  **..................................................**

**Cya_CZS_27J_KY379866**  **..................................................**

**Cya_BS4_AF330252**  **..................................................**

**Cya_CZS_25G_KY379864**  **..................................................**

**Cya_CZS_22C_KY379861**  **..................................................**

**Cya_CZS_19G_KY379860**  **..................................................**

**Cya_CZS_40A_KY379875**  **..................................................**

**Cya_gra_PCC_6307_NR114406** **..................................................**

**Cya_Suigetsu_CG1_AB610890** **..................................................**

**mar_Syn_WH_7803_AF081834**  **..................................................**

**Pro_mar_MIT_9301_NC009091** **....A.............................................**

**S_elong_PCC_6301_NR074309** **......A..U........C....................C..........**

1110 1120 1130 1140 1150

....|....|....|....|....|....|....|....|....|....|

**Cya_CZS_24G_KY379862**  **CAUCAUGCCCCUUACAUCCUGGGCUACACACGUACUACAAUGCUACGGAC**

**'Syn'_WH_5701_AY172832**  **..................................................**

**Cya_CZS_25K_KY379865**  **..................................................**

**Cya_CZS_48M_KY379890**  **..................................................**

**Cya_SAG_3_81_NR125481**  **..................................................**

**Cya_CZS_45I_KY379883**  **..................................................**

**Cya_Suigetsu_CR1**  **..................................................**

**Cya_PCC_7001_AM709626**  **..................................................**

**Cya_CZS_34G_KY379868**  **..................................................**

**Cya_CZS_27J_KY379866**  **..................................................**

**Cya_BS4_AF330252**  **..................................................**

**Cya_CZS_25G_KY379864**  **..................................................**

**Cya_CZS_22C_KY379861**  **..................................................**

**Cya_CZS_19G_KY379860**  **..................................................**

**Cya_CZS_40A_KY379875**  **..................................................**

**Cya_gra_PCC_6307_NR114406** **..................................................**

**Cya_Suigetsu_CG1_AB610890** **..................................................**

**mar_Syn_WH_7803_AF081834**  **..................................................**

**Pro_mar_MIT_9301_NC009091** **................C.................................**

**S_elong_PCC_6301_NR074309** **............................................C.....**

1160 1170 1180 1190 1200

....|....|....|....|....|....|....|....|....|....|

**Cya_CZS_24G_KY379862**  **AAAGGGCAGCAAACUCGCGAGAGCUAGCAAAUCCCAUAAACCGUGGCUCA**

**'Syn'_WH_5701_AY172832**  **..................................................**

**Cya_CZS_25K_KY379865**  **..................................................**

**Cya_CZS_48M_KY379890**  **..................................................**

**Cya_SAG_3_81_NR125481**  **..................................................**

**Cya_CZS_45I_KY379883**  **..................................................**

**Cya_Suigetsu_CR1**  **..................................................**

**Cya_PCC_7001_AM709626**  **..................................................**

**Cya_CZS_34G_KY379868**  **..................................................**

**Cya_CZS_27J_KY379866**  **..................................................**

**Cya_BS4_AF330252**  **..................................................**

**Cya_CZS_25G_KY379864**  **.......U....................U.....................**

**Cya_CZS_22C_KY379861**  **..................................................**

**Cya_CZS_19G_KY379860**  **......UU....G..........U....U.....................**

**Cya_CZS_40A_KY379875**  **......UU....G..........U....U.....................**

**Cya_gra_PCC_6307_NR114406** **......UU....G..........U....U.....................**

**Cya_Suigetsu_CG1_AB610890** **......UU....G..........U....U.....................**

**mar_Syn_WH_7803_AF081834**  **....A.......GU.......GA.A........U................**

**Pro_mar_MIT_9301_NC009091** **..................................................**

**S_elong_PCC_6301_NR074309** **.GC.A.AC..G..GC......GUGA........U.CC......G......**

1210 1220 1230 1240 1250

....|....|....|....|....|....|....|....|....|....|

**Cya_CZS_24G_KY379862**  **GUUCAGAUCGUAGGCUGCAACUCGCCUACGUGAAGGAGGAAUCGCUAGUA**

**'Syn'_WH_5701_AY172832**  **..................................................**

**Cya_CZS_25K_KY379865**  **..................................................**

**Cya_CZS_48M_KY379890**  **..................................................**

**Cya_SAG_3_81_NR125481**  **..................................................**

**Cya_CZS_45I_KY379883**  **..................................................**

**Cya_Suigetsu_CR1**  **..................................................**

**Cya_PCC_7001_AM709626**  **..................................................**

**Cya_CZS_34G_KY379868**  **..................................................**

**Cya_CZS_27J_KY379866**  **..................................................**

**Cya_BS4_AF330252**  **..................................................**

**Cya_CZS_25G_KY379864**  **..................................................**

**Cya_CZS_22C_KY379861**  **..................................................**

**Cya_CZS_19G_KY379860**  **..................................................**

**Cya_CZS_40A_KY379875**  **..................................................**

**Cya_gra_PCC_6307_NR114406** **..................................................**

**Cya_Suigetsu_CG1_AB610890** **..................................................**

**mar_Syn_WH_7803_AF081834**  **..................................................**

**Pro_mar_MIT_9301_NC009091** **...................................U..............**

**S_elong_PCC_6301_NR074309** **........U.C................G.A......C.............**

1260 1270 1280 1290 1300

....|....|....|....|....|....|....|....|....|....|

**Cya_CZS_24G_KY379862**  **AUCGCAGGUCAGCAUACUGCGGUGAAUACGUUCCCGGGCCUUGUACACAC**

**'Syn'_WH_5701_AY172832**  **..................................................**

**Cya_CZS_25K_KY379865**  **..................................................**

**Cya_CZS_48M_KY379890**  **..................................................**

**Cya_SAG_3_81_NR125481**  **..................................................**

**Cya_CZS_45I_KY379883**  **..................................................**

**Cya_Suigetsu_CR1**  **..................................................**

**Cya_PCC_7001_AM709626**  **..................................................**

**Cya_CZS_34G_KY379868**  **..................................................**

**Cya_CZS_27J_KY379866**  **..................................................**

**Cya_BS4_AF330252**  **..................................................**

**Cya_CZS_25G_KY379864**  **..................................................**

**Cya_CZS_22C_KY379861**  **..................................................**

**Cya_CZS_19G_KY379860**  **..................................................**

**Cya_CZS_40A_KY379875**  **..................................................**

**Cya_gra_PCC_6307_NR114406** **..................................................**

**Cya_Suigetsu_CG1_AB610890** **..................................................**

**mar_Syn_WH_7803_AF081834**  **..................................................**

**Pro_mar_MIT_9301_NC009091** **..................................................**

**S_elong_PCC_6301_NR074309** **..................................................**

1310 1320 1330 1340

....|....|....|....|....|....|....|....|..

**Cya_CZS_24G_KY379862**  **CGCCCGUCACACCAUGGAAGUUGGCCAUGCCCGAAGUCGUUA**

**'Syn'_WH_5701_AY172832**  **..........................................**

**Cya_CZS_25K_KY379865**  **..........................................**

**Cya_CZS_48M_KY379890**  **..........................................**

**Cya_SAG_3_81_NR125481**  **...........................C..............**

**Cya_CZS_45I_KY379883**  **...........................C..............**

**Cya_Suigetsu_CR1**  **...........................C........C.....**

**Cya_PCC_7001_AM709626**  **...........................C..............**

**Cya_CZS_34G_KY379868**  **...........................C........C.....**

**Cya_CZS_27J_KY379866**  **...........................C..............**

**Cya_BS4_AF330252**  **...........................C..............**

**Cya_CZS_25G_KY379864**  **...........................C..............**

**Cya_CZS_22C_KY379861**  **...........................C..............**

**Cya_CZS_19G_KY379860**  **..........................................**

**Cya_CZS_40A_KY379875**  **..........................................**

**Cya_gra_PCC_6307_NR114406** **..........................................**

**Cya_Suigetsu_CG1_AB610890** **..........................................**

**mar_Syn_WH_7803_AF081834**  **...........................C........C.....**

**Pro_mar_MIT_9301_NC009091** **..........................................**

**S_elong_PCC_6301_NR074309** **..........................................**

**FIGURES**

**
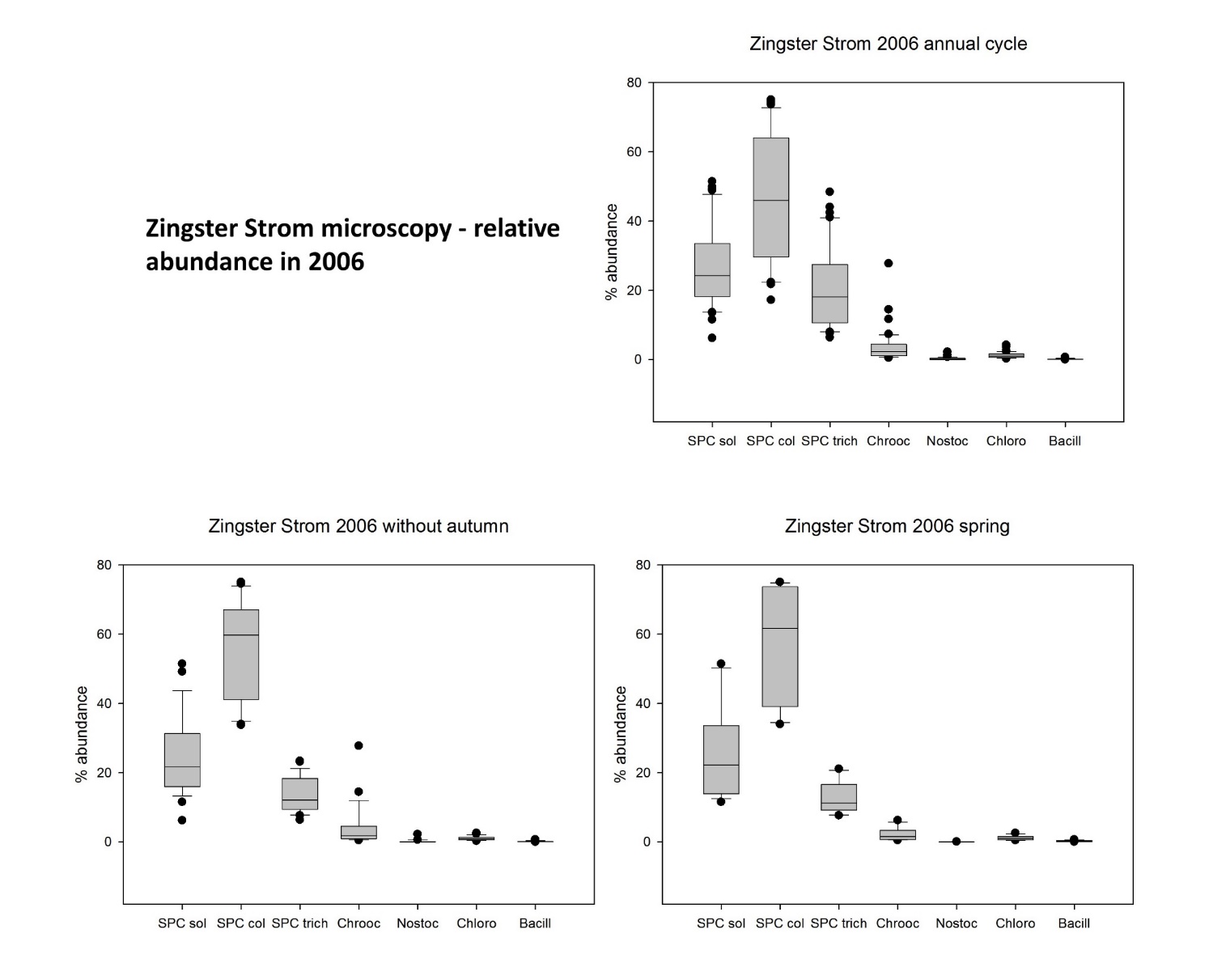
**

**Supplement figure 1** Box-Whisker-Plots of the 2006 relative abundance at Zingster Strom (year-round n=41, year-round without autumn n=28, spring n=14). Plot SPC-sol: Synechococcales solitary cells; SPC-col: Synechococcales colonies; SPC-trich: Synechococcales trichomes; Chrooc.: Chroococcales; Nostoc: Nostocales; Chloro: Chlorophyceae; Bacill: Bacillariophyceae.
 
**
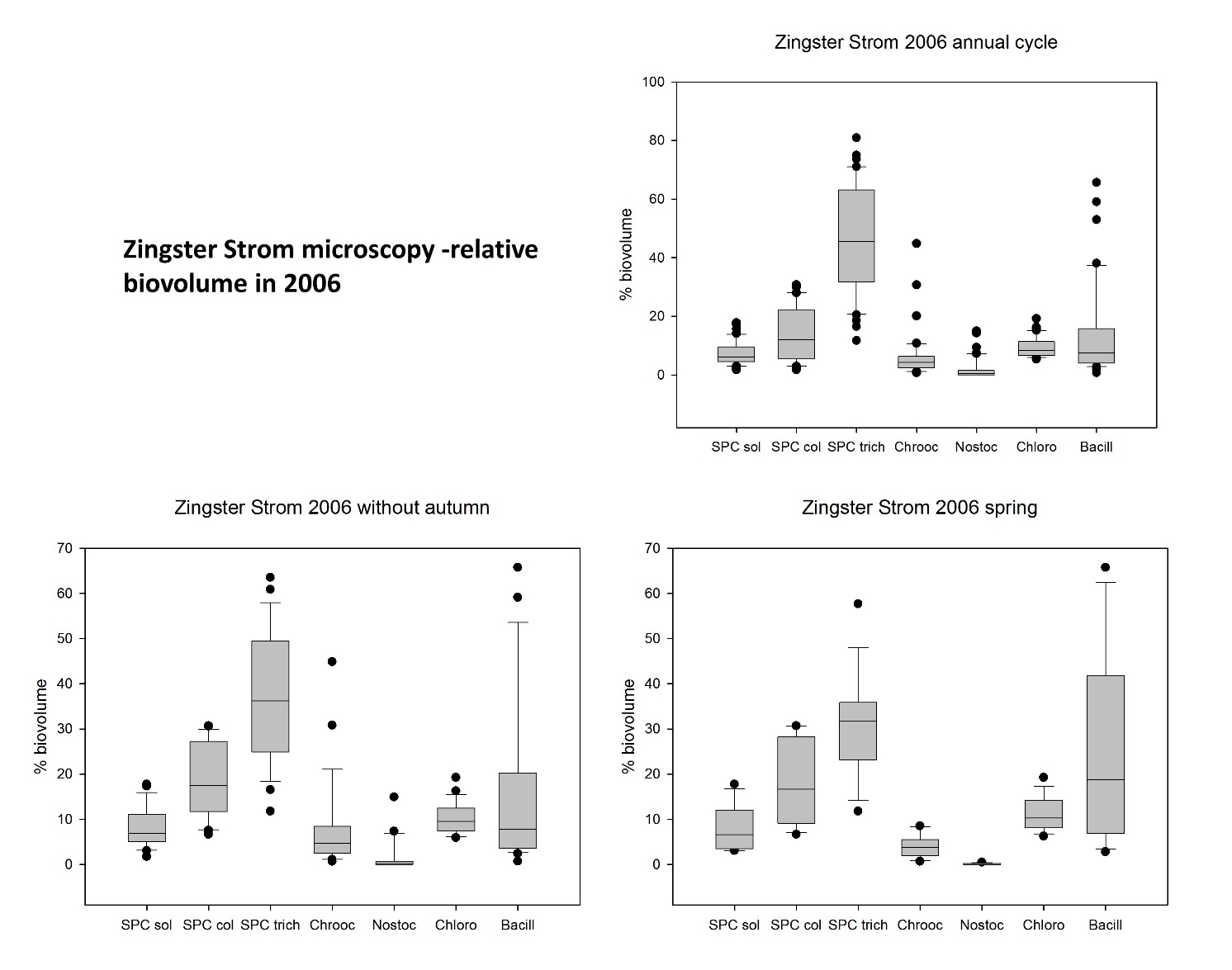
Supplement figure 2** Box-Whisker-Plots of the 2006 relative biovolume at Zingster Strom (year-round n=41, year-round without autumn n=28, spring n=14). SPC-sol: Synechococcales solitary cells; SPC-col: Synechococcales colonies; SPC-trich: Synechococcales trichomes; Chrooc.: Chroococcales; Nostoc: Nostocales; Chloro: Chlorophyceae; Bacill: Bacillariophyceae.
 
**
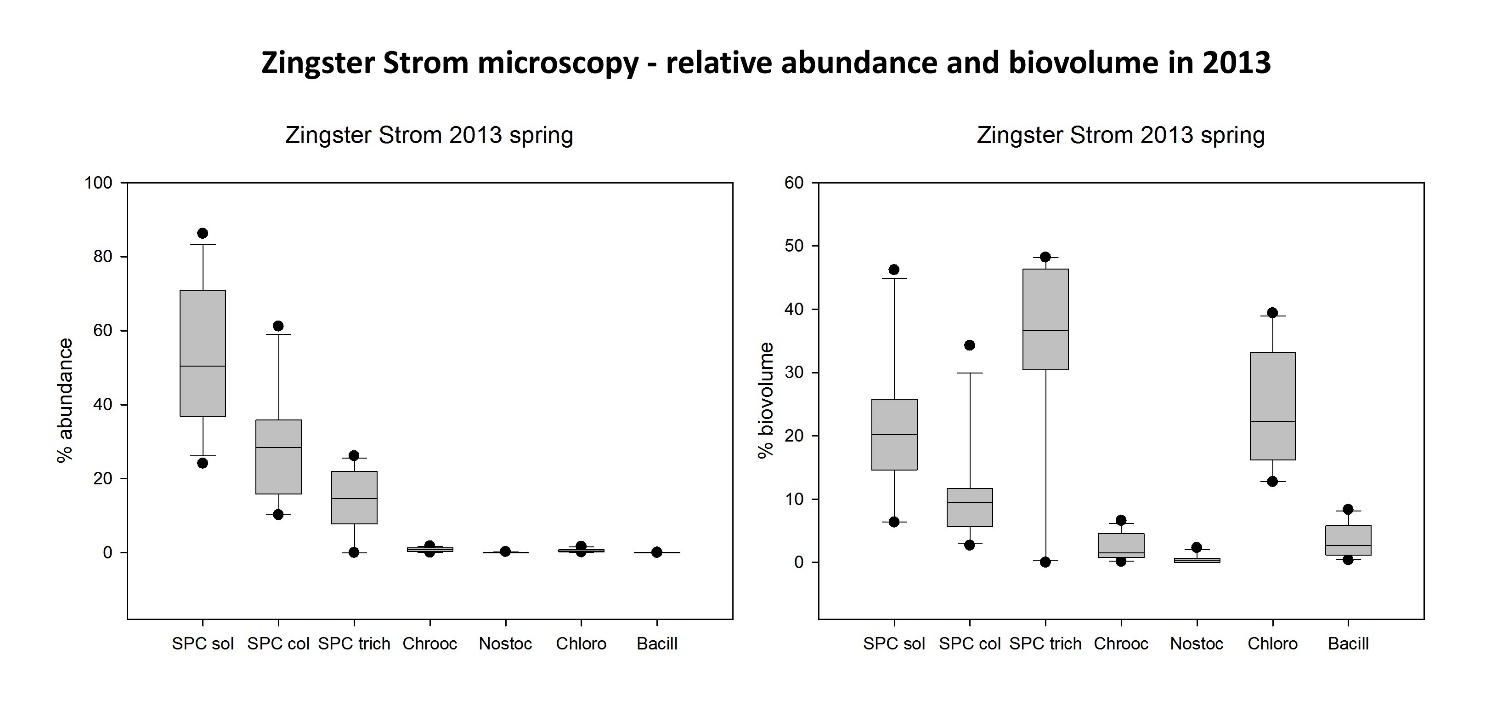
**

**Supplement figure 3** Box-Whisker-Plots of the spring 2013 relative abundance and biovolume at Zingster Strom (n=11). SPC-sol: Synechococcales solitary cells; SPC-col: Synechococcales colonies; SPC-trich: Synechococcales trichomes; Chrooc: Chroococcales; Nostoc: Nostocales; Chloro: Chlorophyceae; Bacill: Bacillariophyceae.
